# Cross-feeding options define genome evolution and community assembly of deep groundwater microbiome

**DOI:** 10.1186/s40793-026-00865-z

**Published:** 2026-02-17

**Authors:** Maryam Rezaei Somee, Carolina González-Rosales, Matti Gralka, Stephanie Turner, Stefan Bertilsson, Mark Dopson, Maliheh Mehrshad

**Affiliations:** 1 Centre for Ecology and Evolution in Microbial Model Systems (EEMiS), Linnaeus University, 391 82 Kalmar, Sweden; 2 Systems Biology Group, Amsterdam Institute for Life and Environment (A-LIFE) and Amsterdam Institute of Molecular and Life Sciences (AIMMS), Vrije Universiteit Amsterdam, Amsterdam, The Netherlands; 3 Department of Aquatic Sciences and Assessment, Science for Life Laboratory, Swedish University of Agricultural Sciences, 750 07 Uppsala, Sweden; 4 Centre for the Environment (CENWIN), Linnaeus University, 39231 Kalmar, Sweden

**Keywords:** Deep groundwater, Genome size, Metabolic cross-feeding, Modular metabolic analyses

## Abstract

**Background:**

Deep groundwaters populated by diverse and active microbes are among the most energy and nutrient-limited ecosystems. Characteristics of this ecosystem (including nutrient and dispersal limitations, low cell densities, and an episodic growth strategy) interactively underpin the so far elusive eco-evolutionary dynamics of its microbiome. Here, we used genome-resolved modular metabolic analyses of disconnected deep groundwater sites in the Fennoscandian Shield to test how eco-evolutionary constraints in these deep groundwater ecosystems shape microbial genome architecture, metabolic versatility, and community assembly at different depths.

**Results:**

The analysis revealed that lineages with larger genomes (≥ 2.6 Mb) maintained higher population sizes in the deepest and most oligotrophic groundwaters, whereas lineages with known metabolic dependencies, such as and DPANN, declined in relative abundance with depth. This pattern was interpreted as consistent with limited opportunities for sustained metabolic cross-feeding in these ecosystems. Moreover, while similar ecological niches based on cross-feeding interactions and potential primary production were available across different boreholes, distinct microbial lineages appeared to occupy these niches at each site.

**Conclusion:**

The findings provided new insights into the role of metabolic cross-feeding in genome evolution and community assembly of deep groundwater microbiomes. By extending the streamlining theory, this study underscores the critical influence of ecological interactions, particularly metabolic exchanges, in shaping microbial life under severe nutrient limitation, offering new insights into subsurface microbial communities.

**Supplementary Information:**

The online version contains supplementary material available at 10.1186/s40793-026-00865-z.

## Background

Deep oligotrophic groundwaters are among the most energy and nutrient-limited ecosystems on the planet. Yet, they sustain diverse and metabolically active communities [1, 2] that encompass representatives from all domains of life, as well as viruses [3, 4]. A deep aquifer here is referred to as fracture-hosted groundwater located tens to hundreds of meters below the surface and hydrologically disconnected from modern recharge [5, 6]. The microbial community composition of deep groundwaters is defined by the available nutrients, electron donors for chemolithotrophic metabolisms (e.g., H_2_, CH_4_, and reduced sulfur plus iron species), and geochemical features of the bedrock hosting groundwaters [7]. Recent studies also reveal the existence of a common “core” microbiome in Fennoscandian Shield deep groundwaters [3] that is mainly shaped by the ecological convergence of species in communities inhabiting similar geologies [3]. Beyond the importance of niche dimensions (both biotic and abiotic) for shaping the composition of deep groundwater communities [3], it is not fully understood how the characteristic features of the deep groundwater affects the eco-evolutionary dynamics of its microbiota.

Despite ongoing debate around the impact of environmental factors in the evolution of genome features (i.e., genome size and GC-content) [8], the assumption that genomes with lower GC-content and smaller genome size can be more abundant in nutrient-limited ecosystems [9] seems to have been confirmed at least for oligotrophic niches in the surface ocean and lakes [10–12]. Abundant lineages with streamlined genomes that dominate these habitats are hypothesized to compensate for auxotrophies and metabolic dependencies via cross-feeding of externally supplied substrates or from tight symbiotic interactions with other community members [13], putting the black queen hypothesis in play for penalizing further loss of essential functions at the level of communities [14]. A well-documented example is *Prochlorococcus*, which has lost the catalase–peroxidase gene (*katG*) and therefore, depends on co-occurring heterotrophic bacteria for hydrogen-peroxide detoxification [14]. This reliance on the community exemplifies how genome reduction can generate metabolic dependencies that are stabilized through cross-feeding. Following the same logic, the lower energy and nutrient demands of lineages with smaller genome sizes and lower GC-content should in theory, allow them to maintain larger populations in the prevailing oligotrophic conditions of deep groundwaters. However, deep groundwater microbiomes show fundamental differences in dispersal mechanisms and symbiosis strategies compared to surface communities. Cell sparsity [13] and episodic growth strategies [3] further restrict nutrient availability and metabolic cross-feeding options, which together could potentially contribute to the emergence of different eco-evolutionary avenues that remain elusive.

Deep groundwater ecosystems in this study represent confined aquifer systems that are largely disconnected from inputs of labile organic matter from the solar-exposed surface of our planet. Compounds able to reach these deep niches (e.g., terrigenous lignin) are barely degradable under the prevailing anaerobic conditions [15]. Thus, conducive carbon acquisition strategies in deep groundwaters are scarce and limited to carbon fixation and recycling of cell debris [16]. Additionally, the main available nitrogen sources are expected to be ammonium (originating from cell necrosis), nitrate (also used as a final electron acceptor), and nitrogen gas (N_2_) [17]. Previous studies suggest that deep oligotrophic groundwater microbes employ an episodic growth strategy to ensure their subsistence [3]. The residual impact of this episodic growth could affect their symbiosis and interdependencies due to interruptions in different metabolic pathways during each growth episode. Consequently, to understand metabolic interactions of the deep groundwater microbiome in the context of predominantly episodic growth, the prevalence of specific modules in carbon and nitrogen acquisition pathways needs to be examined for the production of intermediate compounds potentially relevant for metabolic interactions within the community.

This study used an extensive dataset termed the “Fennoscandian Shield Genomic Database” (FSGD) [3] including metagenome-assembled genomes and single-cell amplified genomes originating from deep oligotrophic groundwaters of the Äspö Hard Rock Laboratory (Äspö HRL) in Sweden and Olkiluoto Island, Finland. They are typically anoxic and characterized by low concentrations of labile organic carbon, with microbial metabolism relying primarily on inorganic electron donors and acceptors such as hydrogen, nitrate, sulfate, and ammonium [3, 17, 18]. Inspecting the genomic features of the microbial community inhabiting each borehole and the metabolic cross-feeding strategies they adopt for subsistence in the deep groundwater ecosystems provided novel clues towards the decisive role of metabolic cross-feeding in the eco-evolutionary dynamics of these microbes adding to the existing understanding of streamlining theory. In this study, we investigated how genome architecture and modular metabolic capacities vary across disconnected deep groundwater sites of the Fennoscandian Shield. Specifically, we aimed to determine how eco-evolutionary constraints in these fracture-bound ecosystems shape microbial genome size, GC-content, and metabolic versatility across depths.

## Results and discussion

### Genome size and GC-content distribution varied in different boreholes

**Fig. 1:**
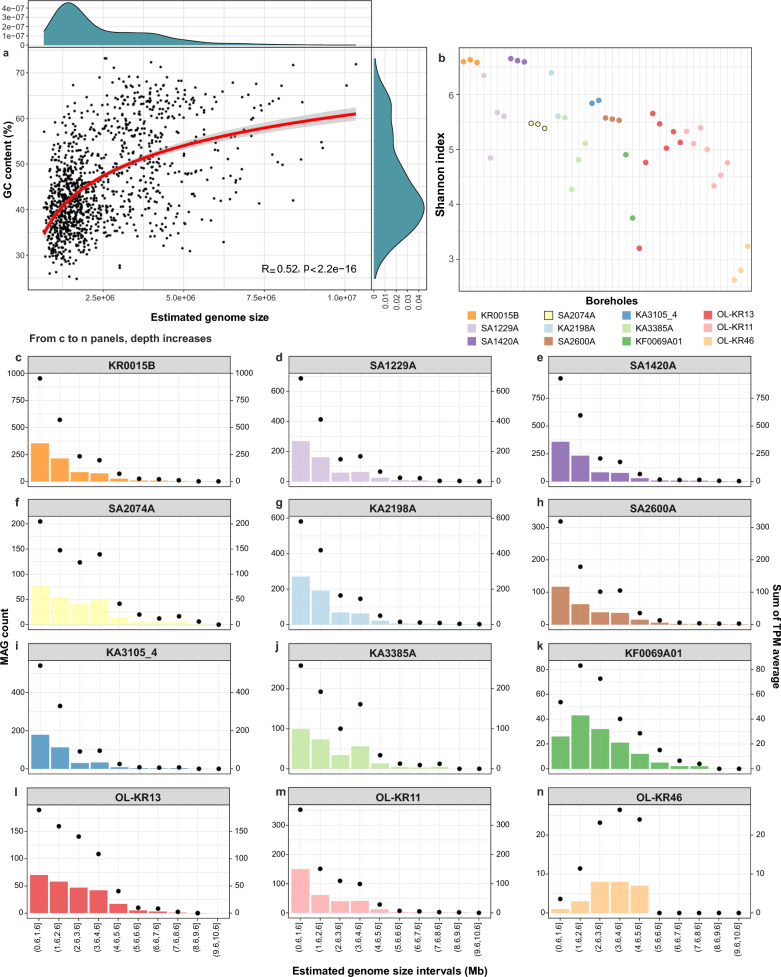
Distribution and prevalence of FSGD MAGs/SAGs in different deep groundwater boreholes. **a** Correlation between GC-content and estimated genome size (EGS) of all representative MAGs/SAGs. Side graphs illustrate the distribution density of MAGs/SAGs across GC and EGS spectrum. **b** Alpha diversity for each borehole calculated based on the Shannon–Wiener index for representative MAGs/SAGs present in each borehole (those with nonzero log_10_ value of the calculated transcript per million (TPM)). **c**–**n** The number of FSGD MAGs/SAGs in 1 Mb intervals along the estimated genome size spectrum are shown as bar plots and are separated in different panels for different boreholes. The overall abundance of MAGs/SAGs in each interval (average of nonzero TPM values in all metagenomes sequenced for each borehole) are overlaid on each panel as dot plots

The FSGD contains 1876 metagenome-assembled genomes (MAGs) and 114 single-cell amplified genomes (SAGs) with ≥ 50% completeness and ≤ 5% contamination. These genomes originated from 43 metagenomes and 114 SAGs, including datasets detailed in Mehrshad et al. [3] plus nine additional sequenced metagenomes (Supplementary Table S1 and Supplementary Fig. S1). Clustering MAGs/SAGs at 95% average nucleotide identity yielded 1,185 representative genome clusters, which were affiliated with 83 phyla and 153 classes (Supplementary Table S2). The MAGs/SAGs GC-content ranged from 25 to 73% and their completeness-corrected estimated genome size (EGS) was in the range of 0.66 to 10.34 MB, with the majority of MAGs/SAGs having an EGS in the range of 1.25–2.5 Mb (Fig. 1a). An overall correlation between the EGS and GC-content was also detected for the FSGD MAGs/SAGs (Fig. 1a). The distribution of MAGs/SAGs along the GC-content range had a major peak at around 40% and two smaller peaks at around 57 and 63% (Fig. 1a). Nevertheless, MAGs/SAGs present in each disconnected borehole showed a different pattern with regards to the overall GC and EGS distribution (Supplementary Figs. S2 and S3). One borehole with strikingly different distribution compared to the overall pattern was the OL-KR46 (Olkiluoto Island, Finland) with MAGs/SAGs present in this borehole (*n* = 27) featuring a GC peak at around 63% (Supplementary Fig. S2) and an EGS peak at around 3.7 Mb (Supplementary Fig. S3). Interestingly, the OL-KR46 borehole intersected the deepest FSGD groundwater at 528.7–531.5 m below surface level (mbsl) and had the lowest Shannon index for alpha diversity (Fig. 1b). It is also among the most saline waters in the dataset (18 ppt; Supplementary Table S1) that highlighted its long residence times and consequently limited connection to surface recharge. Previous work at Olkiluoto shows that the deepest, most isolated fracture zones contain highly evolved saline groundwater with low cell densities and predominantly refractory dissolved organic carbon that is not readily bioavailable, reflecting prolonged geochemical stability and restricted inputs of fresh organic matter [18, 19]. Such physical isolation and long residence times could impact the low alpha diversity observed at OL-KR46 and the enrichment of large-genome, high-GC lineages capable of persisting under chronic energy and nutrient limitations.

The abundance (i.e., population sizes) of representative MAGs with different EGS (grouped in ten categories at 1 Mb intervals) in different boreholes was critical for deciphering eco-evolutionary dynamics of these microbes. Factoring in the abundance of microbes inhabiting each borehole showed that genomes with an EGS between 0.6 to 2.6 Mb had the highest cumulative abundance in all boreholes (Fig. 1c–n) except for KF0069A01 (depth 454.8 mbsl, salinity 24 ppt, two samples, and 143 MAGs) and OL-KR46 (depth 528.7–531.5, salinity 18 ppt, three samples, and 27 MAGs). In these deep fracture systems, elevated salinity mainly reflects long groundwater residence times and hydrological isolation rather than a direct physiological stressor and therefore, serves as an indicator of reduced inputs of fresh organic carbon and electron donors. For KF0069A01, the average of non-zero transcript per million (TPM) abundance of MAGs/SAGs peaked in the 1.6–2.6 Mb EGS range, followed closely by the 2.6–3.6 Mb range (Fig. 1k), whereas in OL-KR46 the highest TPM abundance occurred in the 3.6–4.6 Mb EGS range (Fig. 1n). TPM values reported here represent normalized metagenomic coverage calculated by CoverM and were used as a length- and depth-normalized measure of genome abundance. In marine oligotrophic systems, canonical streamlined lineages, such as *SAR11* and *Prochlorococcus*, typically have very compact genomes (~ 1.3–2.5 Mb), reflecting strong selection for metabolic minimalism under nutrient-poor conditions [20]. However, crystalline bedrock hosted groundwaters in the deepest and most isolated boreholes studied here (KF0069A01 and OL-KR46) harbored lineages with larger genomes (≥ 2.6–4.6 Mb) that persisted and often dominated. This pattern suggested that the balance between the costs and benefits of genome reduction was ecosystem-dependent and that conditions in deep fractured waters do not favor canonical genome streamlining.

MAGs/SAGs detected in the KF0069A01 and OL-KR46 boreholes with an EGS in the range of 0.6 to 1.6 Mb mainly belonged to *Patescibacteria* (11.2 and 3.7% of MAGs/SAGs present in the borehole, respectively) and DPANN (2.1 and 0% of MAGs/SAGs present in the borehole, respectively). The shift in the peak of genome size could be partially attributed to the decreasing prevalence of *Patescibacteria * and DPANN within the community of the deepest boreholes, both at the Äspö HRL and the Olkiluoto locations (Supplementary Fig. S4). This decrease in prevalence could be a consequence of reduced symbiotic encounters in the deeper groundwater, specifically since it is shown that symbiotic interactions are critical for the survival of representatives of these lineages [21, 22].

A correlation between the portion of noncoding DNA in the genome and either genome size (Supplementary Fig. S5**)** or GC-content (Supplementary Fig. S6) was generally weak or insignificant across boreholes. A notable negative correlation was detected only for borehole OL-KR46 (R = -0.68, *p* = 9e-05). This implied that microbes potentially minimized the cost of replication by reducing the noncoding fraction of their genomes. However, the percentage of their non-coding DNA does not drop below 9%, which is still far from the 5% reported for streamlined SAR11 [10] in the ocean.

The population size of representative MAGs/SAGs within each borehole, calculated as an average of their non-zero abundances (log_10_ of TPM value) in all samples of that borehole, showed a normal distribution for most boreholes (Supplementary Fig. S7). There was no strong correlation between the calculated population size and EGS (Supplementary Fig. S8) or the GC-content of MAGs/SAGs in each borehole (Supplementary Fig. S9). This highlighted that lineages from across the range of EGS and GC-content can develop large populations. Further dividing reconstructed MAGs/SAGs into different phyla also detected a range of different population sizes among representatives of each phylum (Supplementary Fig. S10).

### Carbon fixation was more common and through efficient pathways in high GC-content lineages

**Fig. 2:**
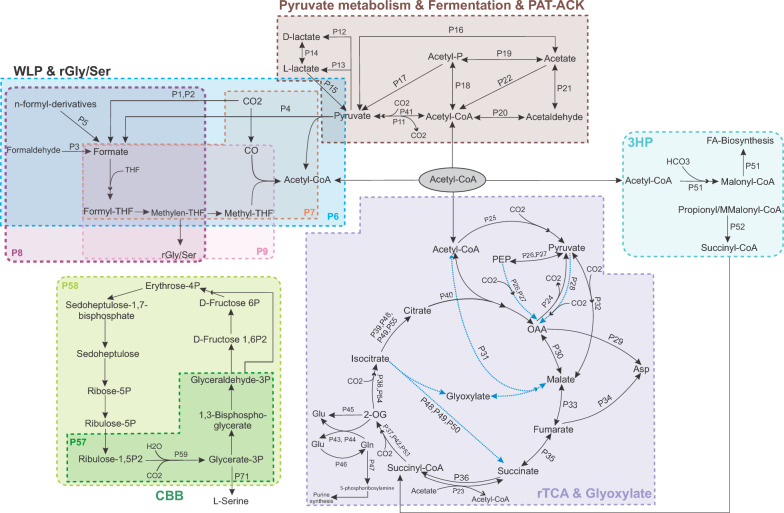
Schematic overview of carbon fixation pathways and designated metabolic modules used in this study. Anaplerotic pathways are shown with gray arrows. Modules related to transporters are not included. Only pathways that were detected in recovered FSGD MAGs/SAGs are represented. KEGG orthologies (KOs) encoding the hydroxy-propionate-hydroxy-butyrate pathway (HPHB) were not detected in FSGD MAGs/SAGs and the pathway is not included. Abbreviations are as follows, PAT-ACK: phosphate acetyltransferase-acetate kinase; WLP & rGly/Ser: Wood-Ljungdahl pathway and reductive glycine/serine; CBB: Calvin-Benson-Bassham cycle, rTCA: reductive tricarboxylic acid/reductive citrate cycle; 3HP: 3-hydroxypropionate bi-cycle

To define the role of metabolic capabilities in eco-evolutionary dynamics of deep groundwater microbiomes, representative FSGD MAGs/SAGs were surveyed for the presence/absence of genes encoding carbon fixation and nitrogen acquisition pathways. To account for their episodic growth in response to the episodic availability of nutrients [3], a modular metabolic analysis was performed. For this approach C-fixation pathways (reductive citrate cycle (rTCA), 3-hydroxy-propionate bi-cycle (3HP), dicarboxylate-hydroxybutyrate, hydroxy-propionate-hydroxy-butylate (HPHB), Calvin-Benson-Bassham cycle (CBB), reductive acetyl-CoA/Wood-Ljungdahl pathway (WLP), and phosphate acetyltransferase-acetate kinase (PAT-ACK)) were broken down to 58 modules based on KEGG (containing 168 genes; Fig. 2). These modules resulted in the production of intermediate compounds that could potentially be used for cross-feeding and therefore, 25 transporters (encoded by 39 genes) specialized for acquisition/export of these intermediates from the milieu were also surveyed. Furthermore, five modules involved in inorganic N-acquisition (ten genes encoding nitrogen fixation, nitrate/nitrite assimilation, nitrate/nitrite dissimilation, plus ten additional genes encoding ammonium permease and NO_3_/NO_2_ transporters) were included (a full list of modules and their enzymes is supplied in Supplementary Table S3).

Genes encoding the enzymes involved in carbon fixation (CO_2_ or HCO_3_^−^) within the rTCA, 3HP, HPHB, and WLP pathways were more prevalent among lineages with higher GC-content and larger EGS except for the gene encoding CBB key enzyme Rubisco (Supplementary Figs. S11 and S12). Here, enzymes catalyzing key carboxylation reactions were focused upon that constitute the biochemical entry points of inorganic carbon into central metabolism. Enzymes such as PEP carboxylase, PEP carboxy kinase (PEPCK), pyruvate carboxylase, crotonyl-CoA carboxylase/reductase, and formate dehydrogenase are widely recognized as central components of carbon fixation pathways and are commonly used as indicators of pathway potential in comparative genomics and biochemistry [23, 24]. In the FSGD dataset, these core carboxylating enzymes previously shown to have a high affinity for capturing carbon under both saturation and low concentrations of CO_2_/HCO_3_^−^ [25] showed a higher prevalence among high GC-content FSGD lineages (Supplementary Fig. S11). This pattern suggested a greater genome-encoded potential for inorganic carbon incorporation in these high GC-content lineages and reflected long-term evolutionary investment in carbon fixation capacity.

WLP, reductive glycine (rGly), and rTCA fixation pathways are energy-efficient in having an ATP requirement of less than 1,1 (per acetyl-CoA), and 3 (per pyruvate), respectively [25, 26]. Among these, the rTCA was more prevalent in high GC-content lineages (Supplementary Fig. S13, panel 3) and while the rTCA does not have the lowest ATP requirement, the anoxic deep groundwater is a conducible environment for its oxygen-sensitive enzymes [27] (e.g., pyruvate synthase, 2OG synthase). Furthermore, reducing agents are available at their highest energy level in anoxic environments and under the same environmental redox conditions, carbon fixation enzymes that use ferredoxin as a reducing agent (such as P25 in rTCA), can push this autotrophic reaction forward more than NAD(P)H types [26], further decreasing the rTCA ATP demand. Most of the rTCA modules (leading to the production of intermediate compounds such as oxaloacetic acid (OAA), 2-oxoglutarate (2OG), and malate) were more prevalent among high GC-content lineages (Supplementary Fig. S13, panel 3). These intermediates are key metabolites that can act as precursors for nucleotide- and amino acid-biosynthesis, which would be in greater demand in high GC-content and larger genomes.

2OG and OAA interconnect carbon and nitrogen metabolism [28, 29] with 2OG being the main precursor for amino acid biosynthesis (glutamate/glutamine) and nitrogen assimilation [29]. Glutamate is generated via either the glutamine synthetase-glutamine oxoglutarate aminotransferase (GS-GOGAT) pathway (P43, P44, and P46 as defined in a modular framework in Fig. 2) or the glutamate dehydrogenase (GDH) pathway (P45; Fig. 2), both of which assimilate ammonium while using 2OG as a carbon backbone [29, 30]. The GDH pathway does not require ATP, but the GS-GOGAT pathway does [31]. However, GS-GOGAT is more efficient under low concentrations of 2OG or ammonium and produces two molecules of glutamate [29, 30]. Both ammonium assimilation pathways were prevalent among high GC-content lineages (Supplementary Fig. S13, panel 3), which can potentially contribute to satisfying their higher nitrogen requirements.

At the interface of amino acids and nucleotide biosynthesis [32], the produced glutamine can be converted to 5-phosphoribosylamine via *purF* (amido-phosphoribosyl transferase, P47), which is channeled towards purine metabolism [33] and this enzyme also showed a higher prevalence among high GC-content lineages (Supplementary Fig. S13, panel 3). Moreover, OAA is a precursor for the biosynthesis of aspartate and pyrimidines [28]. Modules for OAA production (P26, P27, P28) as well as those encoding its conversion to aspartate via ASPDH or aspartate aminotransferase (P29), were more prevalent in high GC-content lineages. Aspartate can also be produced from fumarate (P34), which was less prevalent than P29, yet still more common in high GC-content lineages (Fig. 2 and Supplementary Fig. S13 panel 3).

High GC-content MAGs/SAGs more frequently encoded modules associated with the production and utilization of 2OG and OAA as precursors for amino acid and nucleotide biosynthesis. In parallel, anaplerotic pathways can compensate for such fluctuations in these intermediates by replenishing central carbon flow metabolism [32]. Interestingly, some main anaplerotic pathways [34] that can feed the rTCA cycle (e.g., carboxylation of pyruvate by pyruvate carboxylase (P28) or carboxylation of phosphoenolpyruvate by PEP carboxylase (P27), oxidation of malate to pyruvate by the malic enzyme (P32), and the glyoxylate cycle (P48, P49, 50)) were more prevalent among high GC-content lineages (Supplementary Fig. S13, panel 3). Furthermore, malate as a regulator of the central carbon metabolism [30] can be compensated for by the glyoxylate cycle as an anaplerotic pathway. Modules within the fermentation pathways (P11, P12, P13, P20, P21) could also be connected to rTCA via their potential for producing acetyl CoA (Fig. 2). These modules were also more prevalent among high GC-content lineages (Supplementary Fig. S13, panel 2).

The carbon fixation step of WLP pathway (by formate dehydrogenase (P1 and P2)) or conversion of formaldehyde (P3) or n-formyl derivatives (P5) can lead to the production of formate. Formate can then continue within the WLP pathway (P6, P7) and either be channeled to the reductive glycine/serine (rGly/Ser) pathway (P8, P9: Supplementary Fig. S13, panel 1) or enter the pool of common goods (via passive diffusion [35] or a formate transporter (P60; Supplementary Fig. S13, panel 6) that was present in 21% of formate-producing MAGs/SAGs). If formate is channeled to the rGly/Ser pathway, this will cause production of glycine or serine [36]. Serine is the main C1 pool for biosynthesis of other compounds, including purines and some amino acids (e.g., cysteine, methionine, and tryptophan) [37]. Alternatively, serine can also be produced via the *serB* gene (P10) [37], which was more prevalent among high GC-content FSGD lineages (Supplementary Fig. S13, panel 1). Serine can be converted to pyruvate via L-serine deaminase [38], which was detected in 25.2% of MAGs with rGly/Ser pathway (32 out of 127). Alternatively, glycine can be converted to acetyl-phosphate and then pyruvate using glycine reductase complex [39] (detected in *ca.* 8% of MAGs with rGly pathway).

FSGD MAGs/SAGs only encode the first step of 3HP pathway, producing malonyl-CoA. It is important to note that bicarbonate carboxylating enzymes in carbon fixation pathways preferentially work at substrate saturation levels and are usually active when HCO_3_^−^ is available in high concentrations [26]. Deep groundwaters of the Fennoscandian Shield are characterized by long residence times and hydrochemistry shaped by water–rock interactions, resulting in heterogeneous and temporally variable availability of dissolved inorganic carbon, including bicarbonate [3, 18]. This likely contributed to the lower prevalence of the 3HP pathway in FSGD MAGs/SAGs and hints at potentially low/episodic availability of HCO_3_^−^ in these boreholes. However, the produced malonyl-CoA itself is a main precursor for endogenous fatty acid biosynthesis (P51) using malonyl-CoA:acyl-carrier-protein (ACP) transacylase (fabD, K00645) [40, 41]. Genes involved in endogenous fatty acid biosynthesis were more prevalent among high GC-content lineages. FSGD MAGs/SAGs did not contain the genes needed for converting malonyl-CoA to propionyl-CoA; however, if s-methyl malonyl-CoA is present in their environment, they have the required gene to convert it to succinyl-CoA (P52) and then fumarate or 2OG (P53; Supplementary Fig. S13, panel 4).

Exogenous fatty acid biosynthesis (P56) that is less energy-intensive [42] can follow three paths via the activity of acyl-CoA synthetase (FadD), acyl-ACP synthetase (Aas), or fatty acid kinase (FakAB) to respectively produce acyl-CoA, acyl-ACP, or acyl-phosphate. These compounds are then added to glycerol-3-phospate (G3P) via the activity of PlsB abd (on Acyl-CoA) and PlsXY acyltransferases (on acyl-ACP and acyl-phosphate). Then PlsC catalyzes another addition of acyl-ACP to generate phosphatidic acid, which is a precursor for phospholipids biosynthesis [42]**.** Exogenous fatty acid biosynthesis (P56) was also more prevalent among high GC-content lineages**,** indicating their efficient utilization of all available resources in the environment for different cellular purposes (Supplementary Fig. S13, panel 4).

To gain a more comprehensive perspective on the distribution of metabolic strategies across the communities, the sugar-acid preference (SAP) model [43] was used to predict the FSGD MAGs/SAGs SAP ranges that can extend from 1 (extreme sugar specialists) to -1 (extreme acid specialists). The analysis showed a negative correlation between the SAP index with both GC-content and EGS of FSGD MAGs/SAGs; i.e., a lower SAP index (indicative of acid preference) corresponded to higher GC-content and EGS (Supplementary Fig. S14), potentially attributed to the presence of modules involved in metabolizing intermediates of rTCA (e.g., OAA, 2OG, malate). At the module level, the density of SAP index of MAGs/SAGs for modules associated with acid consumption/production (e.g., P15, P23, P24, P31, P48, P49) and transport (specifically acetate (P79), malate (P74, P78), succinate (P74, P82), and citrate (P80, P83) import/export) were in agreement with the role of these modules in acid metabolism (Supplementary Fig. S15). In all boreholes, the density of the SAP index of MAGs/SAGs peaked around 0.7–0.8 (higher sugar preference); however, in OL-KR46, the peak was at around − 0.5, indicating a higher acid preference of these MAGs. This further confirmed the modular metabolic analyses of this study regarding the importance of metabolizing intermediate acid compounds (i.e., organic acids such as malate, OAA, and 2OG) for lineages inhabiting this borehole.

### Nitrogen acquisition-related genes were more prevalent among higher GC-content lineages

The nitrogen fixation module (PN6), defined by the presence of all core nitrogenase genes (*nifH*, *nifD*, and *nifK*; K02586, K02588, and K02591) was present in 78 out of 1185 FSGD genome clusters (Supplementary Fig. S16) and was also more prevalent among high GC-content lineages. Additionally, high-affinity nitrate/nitrite transporters (module PN1) as well as genes involved in reducing nitrate/nitrite to ammonium (modules PN2, PN3) and ammonium transporter protein (AMT, K03320)/permease (module PN7) were more prevalent among high-GC lineages. Higher GC-content means higher nitrogen requirements since guanine and cytosine pairs require one more nitrogen than adenine and thymidine pairs, which could potentially explain the higher prevalence of different nitrogen uptake and fixation strategies in lineages with higher GC-content (Supplementary Fig. S16).

Taken together, the metabolic profiles of high-GC, larger-EGS lineages supported their role as metabolically versatile generalists capable of flexibly routing carbon through anaplerotic, fermentation, and fatty-acid biosynthesis modules to produce intermediates that can function as public goods. In contrast, ultra-small, low-EGS lineages such as *Patescibacteria * and DPANN encoded truncated central metabolism and relied heavily on transporters and episymbiotic interactions, consistent with specialist, interaction-dependent lifestyles [44]. In surface pelagic systems, streamlined genomes are often associated with abundant specialists that rely on dense interaction networks and externally supplied metabolites [10]. In the deep groundwaters studied here, the opposite association emerges. Metabolically versatile generalists tend to have larger, high-GC genomes that retain broad repertoires for carbon and nitrogen acquisition as well as anaplerotic reactions within a single genome, even at a higher replication and maintenance cost [45]. Conversely, interaction-dependent specialists were progressively filtered out with depth as encounter rates and cross-feeding opportunities likely declined**.**

### Shared metabolic networks in different boreholes offer similar niches that were filled by different lineages

**Fig. 3:**
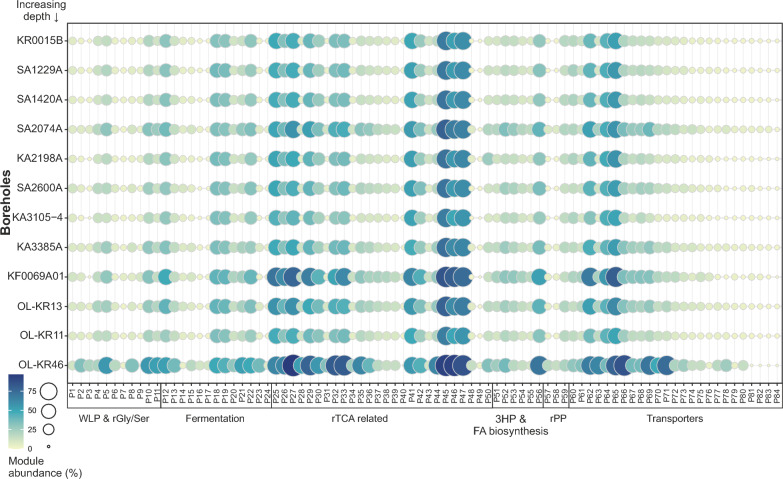
Prevalence of metabolic modules in different boreholes. The size and color of circles indicate the percentage of members within each borehole possessing a corresponding metabolic module. Boreholes are arranged based on their depth from the top to the bottom of the plot

Communities of different boreholes represented a similar distribution profile for different metabolic modules (Fig. 3). This implied that similar metabolic niches were available in the different boreholes, but those lineages with metabolic capabilities that can occupy these niches and the range of their genome GC-content varied across the different boreholes (Supplementary Figs. S17 and S18).

**Fig. 4:**
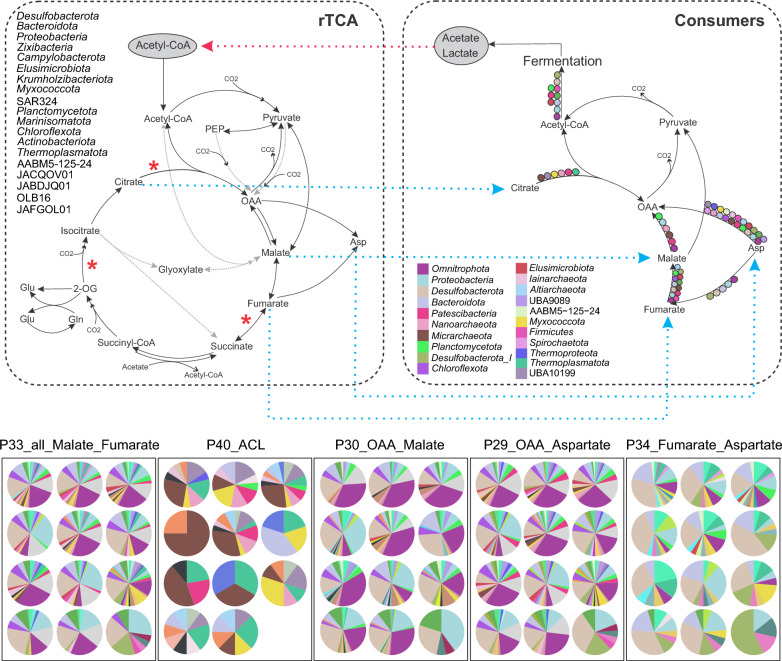
Metabolic cross-feeding among microbial communities of different boreholes. Top left panel shows the complete rTCA cycle, highlighting phyla encoding at least two of the three genes critical for rTCA to operate in the carbon fixation direction (denoted by an asterisk). The top right panel shows the truncated TCA pathway detected in consumers. The main phyla encoding each module are represented in circles with the color legend included in the bottom left side of the panel. Blue dashed arrows show the potential transfer of metabolic products from primary producers to consumers. The red dashed arrow indicates the potential reciprocal utilization of products by primary producers. Bottom panel denotes the composition of microbes carrying each of the metabolic modules present in the consumers in this cross feeding in different boreholes as pie charts. In each subpanel, boreholes are arranged from left to right based on their depth. In bottom subpanels, the order of boreholes from left to right is based on depth as follows; Row 1: KR0015B, SA1420A, SA2074A; Row 2: KA2198A, SA1229A, KF0069A01; Row 3: SA2600A, KA3385A, KA3105-4; Row 4: KR-11, KR-13, KR-46

For the entire TCA cycle to run in the carbon fixation direction (rTCA) the enzymes; fumarate reductase (P35), 2-oxoglutarate: ferredoxin oxidoreductase (2OGOR or *korABCD* respectively modules P37 and P42), and ATP citrate lyase (ACL, module P40), must be present [46] (Fig. 4). Taking this into account, only eight FSGD MAGs/SAGs (affiliated to *Campylobacterota*, *Bacteroidota*, *Myxococcota*, *Desulfobacterota*, and *Thermoplasmatota* phyla) encoded the complete set of genes to carry out the rTCA within a single genome. However, none of these lineages were detected in boreholes OL-KR46 and KA3385A (Supplementary Table S4). There were 113 additional MAGs that carried two of the three necessary enzymes (P35, P37). This could be either due to MAG incompleteness or that they are only performing carbon fixation up to the production of citrate. These lineages were affiliated with *Desulfobacterota*, *Proteobacteria*, *Bacteroidota*, *Campylobacterota*, *Zixibacteria*, *Elusimicrobiota*, *Myxococcota*, *Krumholzibacteriota*, *Planctomycetota*, SAR324, *Thermoplasmatota*, AABM5-125–24, *Actinobacteriota*, *Chloroflexota*, *Marinisomatota*, JABDJQ01, JACQOV01, OLB16, and JAFGOL01 phyla (Fig. 4, Supplementary Figs. S17, S19 and Table S4). From these MAGs, four were present in the OL-KR46 borehole (affiliated to *Desulfobacterota* and *Actinobacteriota*). The GC-content of lineages carrying rTCA-specific genes ranged from 30.88 to 68.26 and their EGS ranged from 2 to 10 Mb (Supplementary Table S4). However, from across the range of GC-content and EGS, these primary-producer lineages can develop large population sizes (Supplementary Fig. S19).

Based on the genomic potential inferred from the analyzed modules, many of the studied modules lead to the production of intermediate compounds such as malonyl-CoA, OAA, 2OG, and fumarate. These intermediates can either be channeled to different cellular pathways (e.g., biosynthesis of fatty acids, amino acids, and nucleotides) or under certain physiological conditions, be released to the environment either by diffusion through the cell membrane or via specialized transporters (for compounds overproduced in the cell or those that cannot be further metabolized). If these compounds were to be released into the environment, they could potentially become a part of a public goods pool, potentially contributing to promiscuous metabolic cross-feeding in the deep oligotrophic groundwater [47].

Even if MAGs/SAGs carried only few enzymes relevant for metabolizing some of these intermediates (rather than featuring the complete pathway), they can contribute to the flow of carbon within and between cells. For example, MAGs/SAGs carrying modules P25 to P33 (i.e., a truncated TCA cycle) in each borehole could take up and metabolize fumarate (as an example of a public good) and even transform it to acetyl-CoA (in reverse from module P33 towards P25). These MAGs/SAGs (*n* = 367) were mainly affiliated with *Omnitrophota*, *Proteobacteria*, *Desulfobacterota*, *Bacteroidota*, *Chloroflexota*, *Planctomycetota*, *Actinobacteriota*, *Micrarchaeota*, *Patescibacteria*, and *Nanoarchaeota* phyla (Supplementary Fig. S17 and Fig. 4). A number of these MAGs/SAGs (*n* = 190) also carried genes for PAT-ACK (P18 and P19; specifically, *n* = 64 belonged to phylum *Omnitrophota*), which further converts acetyl-CoA into acetate while also generating one ATP. The produced acetate could be released to the surrounding environment via the formate transporter (P60) [48] that was present in 51 of these 190 fermenter lineages (Supplementary Fig. S18), with 22 of them belonging to the phylum *Omnitrophota*. Acetate, in turn, can be taken up by primary producers and transformed into acetyl-CoA (via P22 or P23) to replenish the rTCA pathway in an intricate and promiscuous community-level cross-feeding or it can be turned into pyruvate (P16), which feeds the rTCA (Supplementary Fig. S17).

Representatives from *Patescibacteria* (*Portnoybacterales*, BM507, SG8-24, JAHISY01, UBA1369), *Nanoarchaeota* (*Pacearchaeales*, *Woesearchaeales*), and *Micrarchaeota* (*Norongarragalinales*, *Anstonellales*, JACRGF01) encoded the genomic potential to utilize promiscuous transporters (e.g., P64, P65) to take up malate from the pool of putative “public goods” and convert it to pyruvate via malate dehydrogenase (P32) while also conserving reducing power by producing NADPH [49] (Supplementary Fig. S17). 21.7% of the lineages encoding enzymes for P64/P65 are affiliated with *Patescibacteria*. Lineages carrying P32 also encoded the capacity to further metabolize pyruvate into fermentation products such as D-lactate/L-lactate (P12, P13; Supplementary Fig. S17), which could be released into the environment. Interestingly, 34 of the primary producer lineages encoding the rTCA cycle harbor genes to utilize lactate via lactate dehydrogenase (LldEFG [50], P15; Supplementary Fig. S17), thereby converting lactate back to pyruvate and potentially feeding into the rTCA cycle. This community-level network of reciprocal metabolic cross-feeding was detected as genomic potential in all boreholes, although at different prevalence and in different microbial lineages (Fig. 4, Supplementary Figs. S17 and S18).

Apart from this main network of metabolic cross-feeding around intermediates of the rTCA pathway, some other rTCA pathway intermediates can shape peripheral cross-feeding nodes. For example, most MAGs/SAGs with rTCA did not contain ACL (P40) to regenerate the substrates of the cycle. However, they can release citrate to the surrounding environment, which can be taken up by ACL-bearing MAGs and be converted to acetyl-CoA and OAA. This potential was mainly present in representatives of *Micarchaeota*, UBA10199, *Nanoarchaeota*, *Thermoplasmatota*, *Myxococcota*, *Patescibacteria*, *Undinarchaeota*, and *Altiarchaeota* (Fig. 4 and Supplementary Fig. S17). However, ACL was not detected in any of the OL-KR46 MAGs.

Some MAGs/SAGs encoded the capacity to take up aspartate from a putative transient pool of common goods and convert it to malate/fumarate (P29/P34; *n* = 422), which could subsequently be channeled towards pyruvate production. These lineages mainly belonged to *Omnitrophota* (Koll11), *Proteobacteria*, *Desulfobacterota*, *Planctomycetota*, *Patescibacteria*, *Bacteroidota*, *Chloroflexota*, *Micrarchaeota*, and *Nanoarchaeota* phyla. Moreover, these MAGs/SAGs can carry out fermentation (P18 and P19) and provide acetate to the community (*n* = 214; Fig. 4 and Supplementary Fig. S17). Overall, bidirectional interactions are shown to be more prevalent under anoxic conditions, where they enable the community to survive in nutrient-poor environments [47].

Finally, the 3HP pathway provides intermediates that can be directed toward FA biosynthesis (P51). Genes encoding this module were present among representatives of *Proteobacteria*, *Bacteroidota*, *Planctomycetota*, *Zixibacteria*, *Firmicutes*, AABM5-125-24, *Omnitrophota* (Koll11), and *Elusimicrobiota* (Supplementary Fig. S17). Moreover, 62 of the 170 lineages encoding this module also encoded genes for fatty acid export (using AcrAB-TolC complex or FarE [51], P63; Supplementary Fig. S17) and were detected in all boreholes. These lineages can potentially supply fatty acids to CPR representatives that were not capable of synthesizing fatty acids. Surveyed FSGD lineages did not encode the full 3HP pathway. They only contained acetyl-CoA carboxylase that produces malonyl-CoA, which is then supplied to the fatty acid biosynthesis.

In deep crystalline bedrock groundwaters, where fracture systems are physically isolated and energy inputs are extremely restricted, environmental filtering and limited dispersal are expected to shape the community assembly process. Despite differences in the lineages performing these functions, the strong similarity in metabolic niches across boreholes aligns with this view. Comparable redox conditions and shared rTCA-derived intermediates selected for recurrent metabolic modules, while restricted dispersal allowed different taxa to occupy equivalent niches in different sites. This combination of functional convergence with taxonomic turnover was consistent with environmental filtering operating within a dispersal-limited regional pool. Similar assembly patterns are observed in groundwater systems globally. National-scale surveys of New Zealand groundwater wells, spanning a range of aquifer types, depths, and chemistries, show that bacterial community structure correlates strongly with groundwater chemistry, redox potential, and aquifer characteristics [52]. Although these sedimentary aquifers differ from the deep crystalline systems studied here, they reinforce the broader principle that hydrochemical context strongly shapes groundwater microbiomes. In Germany, oligotrophic groundwater hosts low-DOC microbial assemblages in which *Patescibacteria* frequently dominate, reflecting niche specialization under carbon limitation [53]. More broadly, a recent global synthesis of terrestrial deep subsurface microbiomes, including those from the Fennoscandian Shield, Canada, the United States, South Africa, Spain, Switzerland, Russia, and Japan identified recurrent core lineages and functionally coherent metabolic traits across geographically and geologically distinct fracture aquifers [54]. Within this context, the observation that analogous cross-feeding networks recured across disconnected boreholes, yet were reconstructed by different combinations of high-GC and metabolically versatile lineages reflected assembly dominated by trait-based selection operating under severe dispersal limitations. The depth-associated decline of highly interaction-dependent specialists such as *Patescibacteria* and DPANN further suggested that opportunities for metabolic interdependence become increasingly constrained with depth, while larger-genome generalists maintained a resilient metabolic backbone under extreme energy limitation. Borehole OL-KR46 represented an endpoint of this pattern. Extreme oligotrophy, long residence time and fracture isolation intensified environmental filtering and restricted opportunities for metabolic interdependence. In such settings, broad metabolic repertoires were advantageous, consistent with the dominance of larger, high-GC generalists and the reduced prevalence of interaction-dependent specialists at this site (Fig. 1b, k–n). These trends were also visible in the cross-feeding network, which in OL-KR46 was supported by a reduced set of metabolically versatile taxa despite preserving the same overall metabolic architecture (Figs. 3 and 4). Similar combinations of niche selection and dispersal limitation are reported from other hard-rock aquifers [55], and metabolic versatility is shown to increase fitness under energetic stress [56]. Together, these observations provided an eco-evolutionary explanation for why OL-KR46 differed from other boreholes in this study. Additional sampling of deep, hydrologically disconnected groundwaters across diverse geological settings will be critical for testing the generality of these patterns and for refining our understanding of how physical isolation and long-term energy limitation shape microbial genome architecture and community assembly.

## Conclusion

Variation in inferred metabolic cross-feeding potential was observed across deep groundwater environments, which may be associated with eco-evolutionary trajectories of microbes in deeper groundwater. The prevalence of lineages such as DPANN and *Patescibacteria* with patchy metabolism that are most often reliant on symbiotic relations, was lower in deeper and more oligotrophic groundwaters. Furthermore, lineages with larger genome sizes (and higher GC-content) appeared to have larger relative abundances in such ecosystems. Detailed analyses of metabolic modules in different boreholes showed that similar niches were available not only for primary producers but also for putative coupled cross-feeding networks. However, different populations filled these shared niches in different boreholes. The study highlighted a potential role of metabolic cross-feeding in genome evolution and community assembly in deep oligotrophic groundwaters and advances our understanding of the complex interactions and evolutionary pressures in these largely hidden ecosystems on our planet. The results further emphasized that mainstream theories need to be carefully vetted before being applied to new ecosystems with potentially different eco-evolutionary constraints.

## Methods

### Fennoscandian shield genomic database

An extensive dataset consisting of 43 metagenomes and 114 SAGs (Supplementary Table S3) [3] from carbon and energy-limited deep groundwater samples collected from the Swedish Nuclear Fuel and Waste Management Company (SKB) Äspö Hard Rock Laboratory (Äspö HRL) in Sweden and drillholes in Olkiluoto Island, Finland operated by Posiva Oy was used in this study. The metagenomes originated from samples collected from nine boreholes in Äspö HRL (KR0015B, SA1229A, SA1420A, SA2074A, KA2198A, SA2600A, KA3105-4, KA3385A, KF0069A01) and three drillholes in Olkiluoto Island (OL-KR13, OL-KR11, OL-KR46). Samples were collected from different depths in the range of 70 to 528 mbsl (details and metadata for all metagenomes are shown in Supplementary Table S1).

Metagenome-assembled genomes (MAGs) were reconstructed following the method described by Mehrshad et al. [3]. Briefly, the metagenomic sequences were quality-checked and trimmed using Trimmomatic (version 0.36) [57]. The Illumina TruSeq adapter was trimmed based on specific parameters (‘TruSeq3-PE-2.fa:2:30:15 LEADING:3 TRAILING:3 SLIDINGWINDOW:4:15 MINLEN:31’). Each dataset was then individually assembled using MEGAHIT (version 1.1) [58] with customized settings (–k-min 21 –k-max 141 –k-step 12 –min-count 2). Following assembly, contigs of at least 2 kb in length were automatically binned using MetaBat2 [59] with its default settings.

Genome-resolved analyses of these metagenomes and single-cell amplified genomes resulted in the reconstruction of a total of 1990 MAGs/SAGs with ≥ 50% completeness and ≤ 5% contamination [60] according to assessment by CheckM (v.1.2.0) [61]. This database of MAGs/SAGs reconstructed from these metagenomes and used in this study is referred to as the “Fennoscandian Shield Genomic Database” (FSGD for short). The taxonomic affiliation of reconstructed MAGs/SAGs was assigned using GTDB-tk v2.1.0 (reference database R207) [62].

### MAGs/SAGs abundance calculation in different metagenomes

All MAGs and SAGs were further clustered into mOTUs (metagenomic operational taxonomic units) using mOTUlizer (v.0.3.2) at the 95% average nucleotide identity (ANI) threshold [63]. Representative MAGs or SAGs of each mOTU were used for abundance calculation. The abundance of representative MAGs/SAGs in each metagenome was calculated with CoverM (v.0.6.1) using TPM as the normalization method (https://github.com/wwood/CoverM)(List of MAGs/SAGs is provided in Supplementary Table S2).

### Functional annotation of reconstructed MAGs/SAGs and modular metabolic analyses

FSGD MAGs and SAGs were annotated using PROKKA (v.1.12) [64] and then functions were assigned using eggNOG-mapper (v.2.0.15) [65]. In the next step, the presence/absence of genes involved in six prevalent carbon fixation pathways (i.e., reductive citrate cycle (rTCA), reductive acetyl-CoA/Wood-Ljungdahl pathway (WLP), phosphate acetyltransferase-acetate kinase (PAT-ACK), Calvin-Benson-Bassham cycle/reductive pentose phosphate cycle (rPP, rPP-Calvin), 3-hydroxypropionate bi-cycle (3HP) and dicarboxylate-hydroxybutyrate cycle) in different MAGs/SAGs was surveyed using a modular approach as explained below. Additionally, the presence/absence of genes involved in nitrogen metabolism were also surveyed.

To gain a better insight into how these pathways affect the microbial community and their metabolic interdependencies, the C-fixation and nitrogen acquisition pathways were subdivided into modules that lead to the production of intermediate compounds (e.g., formate, pyruvate, oxaloacetate, etc.; Fig. 2). The list of KEGG orthologs (KOs) for genes involved in each module is provided in Supplementary Table S3. In total, 58 carbon fixation-related modules and 25 transporter genes were inspected. Similarly, modules involved in nitrogen acquisition and assimilation and their related transporters were investigated (Supplementary Table S3).

The prevalence of these modules/genes was analyzed in the range of genome size and genome GC-content. In order to normalize the data for comparative analyses, the GC-content range (25–75%) of FSGD MAGs/SAGs was divided into five intervals and the prevalence of each module/gene was calculated by dividing the number of MAGs containing the module/gene in each interval by the total number of MAGs/SAGs in that interval. The same approach was applied for genome size by dividing the range of genome size (0.6–10.3 mb) into ten equal intervals.

### Sugar-acid preference (SAP) analyses

To predict the SAP for MAGs, the procedure outlined in Gralka et al. [43] was followed. Briefly, the total abundance (normalized by the number of genes) of a list (given in the supplementary information) [43] of sugar (*S*) and acid (*A*) genes was computed. The relative abundances of S and A were then used to predict SAP as tanh(s*S* + a*A*), with s = 60.76 and a = − 20.21.

## Supplementary Information

Additional file1 (XLSX 88 KB)

Additional file2 (XLSX 584 KB)

Additional file3 (XLSX 21 KB)

Additional file4 (XLSX 22 KB)

Additional file5 (DOCX 7735 KB)

## Data Availability

Previously released FSGD MAGs can be accessed through the NCBI BioProject under the accession number PRJNA627556. SAGs are publicly available in figshare with the identifier 10.6084/m9.figshare.12170313 under the project name ‘Fennoscandian Shield genomic database (FSGD)’. The additional MAGs used for this study are deposited under the BioProject accession number PRJNA1023754, and their accession numbers are given in Supplementary Table S1.
