# Supplementary material for: Cross-feeding options define genome evolution and community assembly of deep groundwater microbiome
Source: Environ Microbiome. 2026 Feb 17;21:44. doi: 10.1186/s40793-026-00865-z (PMC13014828; doi:10.1186/s40793-026-00865-z)
Supplement: Supplementary file 5 — Additional file5 (DOCX 7735 KB) [file 40793_2026_865_MOESM5_ESM.docx]

**Supplementary Figures:**

**
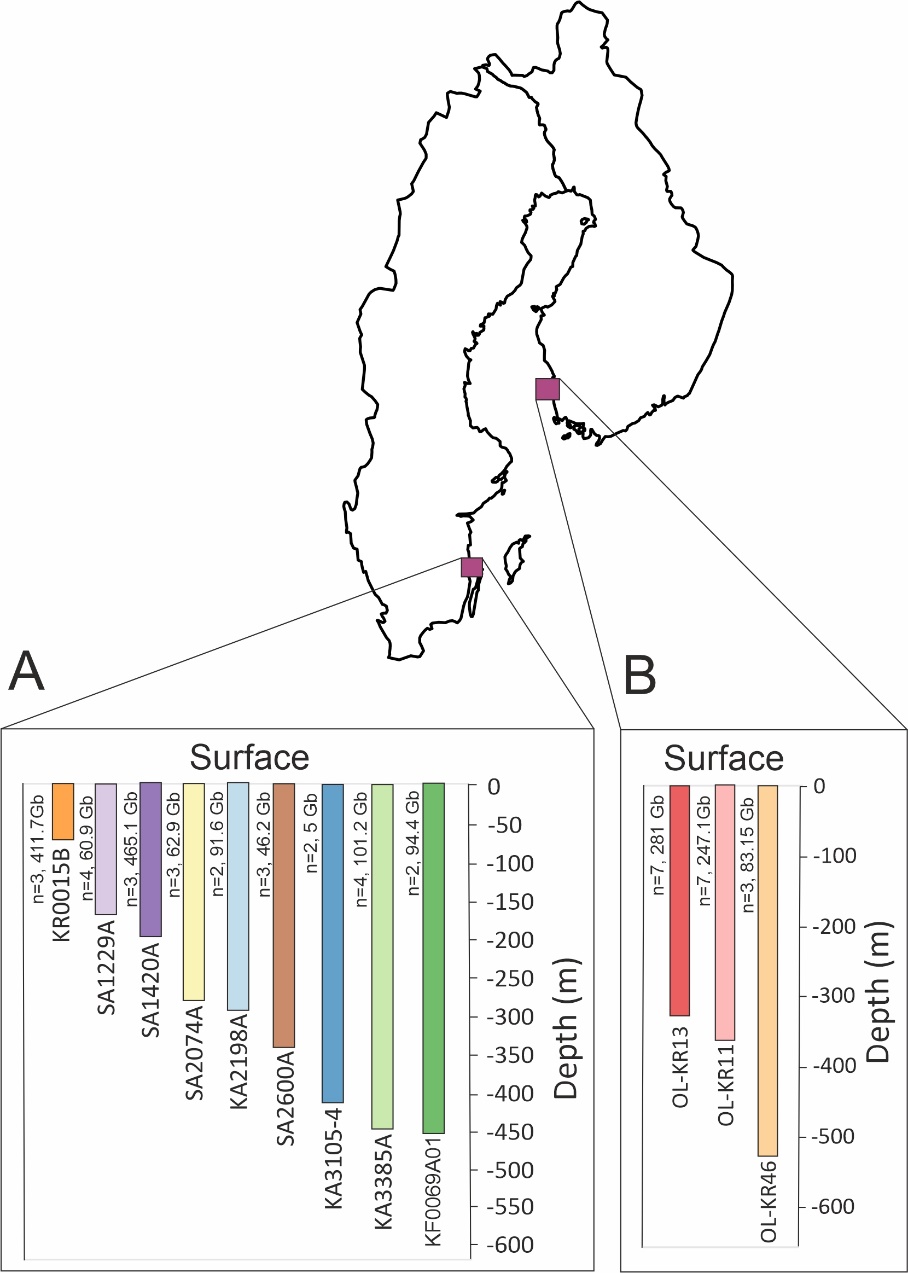
**

**Supplementary Figure S1:** Geographic location and depth distribution of deep subsurface boreholes at the two study sites. A) Äspö Hard Rock Laboratory (Sweden), illustrating the vertical distribution of multiple boreholes spanning shallow to deep intervals from left to right. B) Olkiluoto (Finland), illustrating fewer but deeper boreholes. Depths are shown relative to the surface (0 m), increasing downward. The numbers shown adjacent to each bar denote the number of metagenomic samples (*n*) and the total sequencing yield (Gb) aggregated across all samples from the respective borehole. Boreholes are color-coded consistent with Figure 1.

**
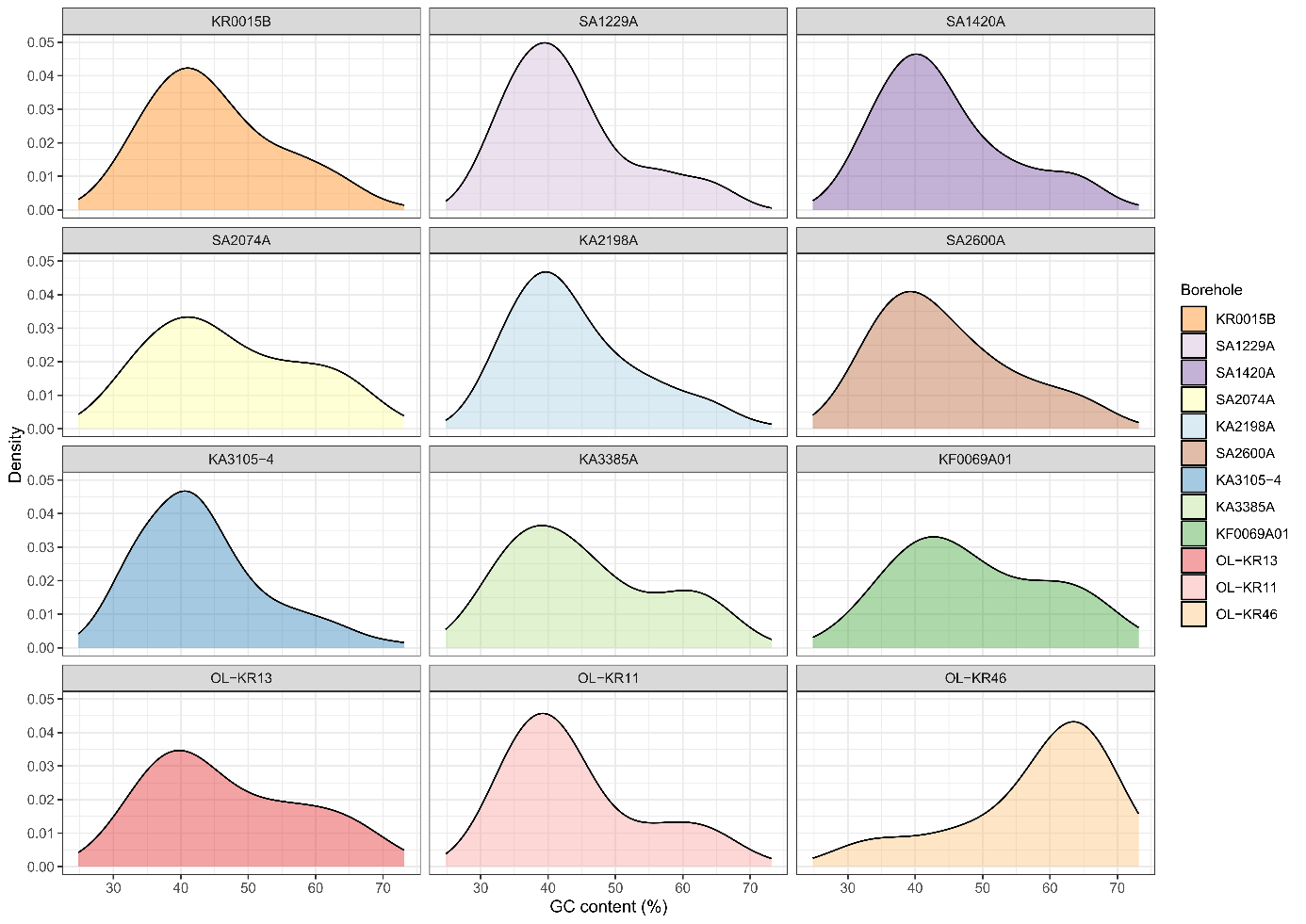
**

**Supplementary Figure S2:** GC density of representative MAGs/SAGs present in different boreholes. MAGs/SAGs with nonzero log_10_ value of the calculated transcript per million (TPM) were considered as present. Panels are ordered from left to right according to increasing sampling depth. Boreholes are color-coded consistently with Figure 1.

**
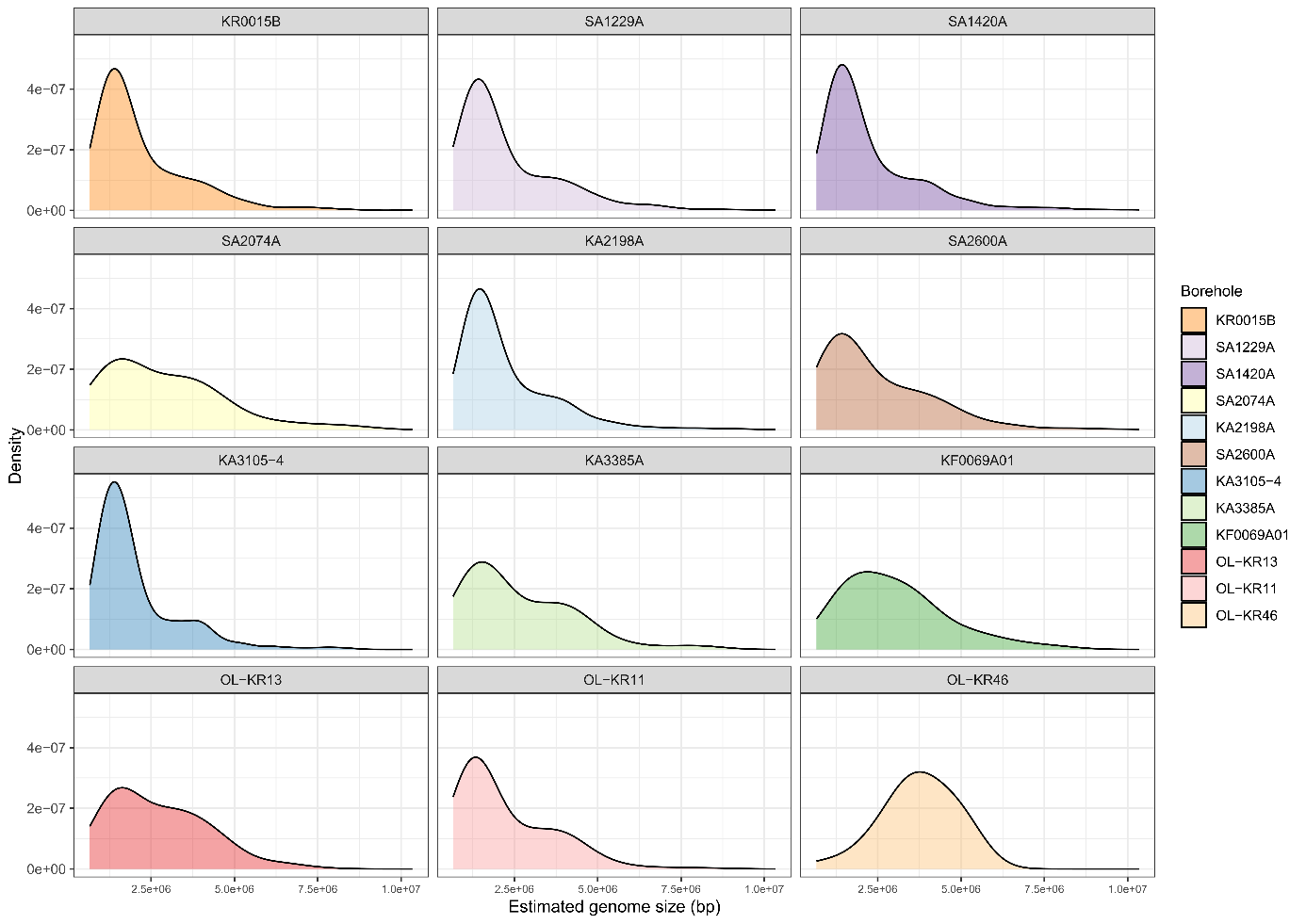
**

**Supplementary Figure S3:** Estimated genome size (EGS) density of representative MAGs/SAGs present in different boreholes. MAGs/SAGs with nonzero log_10_ value of the calculated transcript per million (TPM) were considered as present. Panels are ordered from left to right according to increasing sampling depth. Boreholes are color-coded consistently with Figure 1.

**
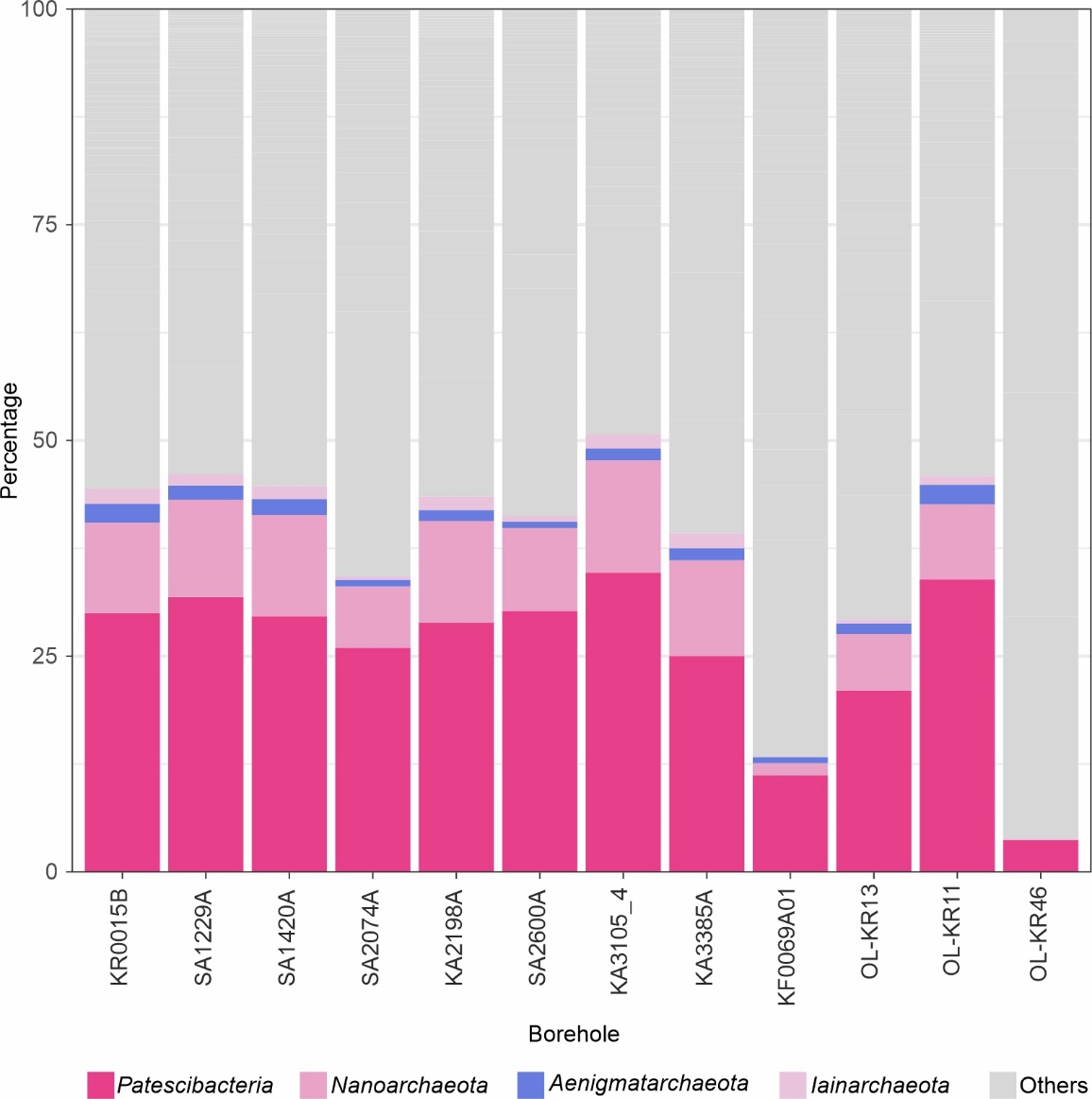
**

**Supplementary Figure S4:** Percentage of MAGs/SAGs affiliated to DPANN superphylum and Patescibacteria phylum among MAGs/SAGs present in different boreholes. Panels are ordered from left to right according to increasing sampling depth.

**
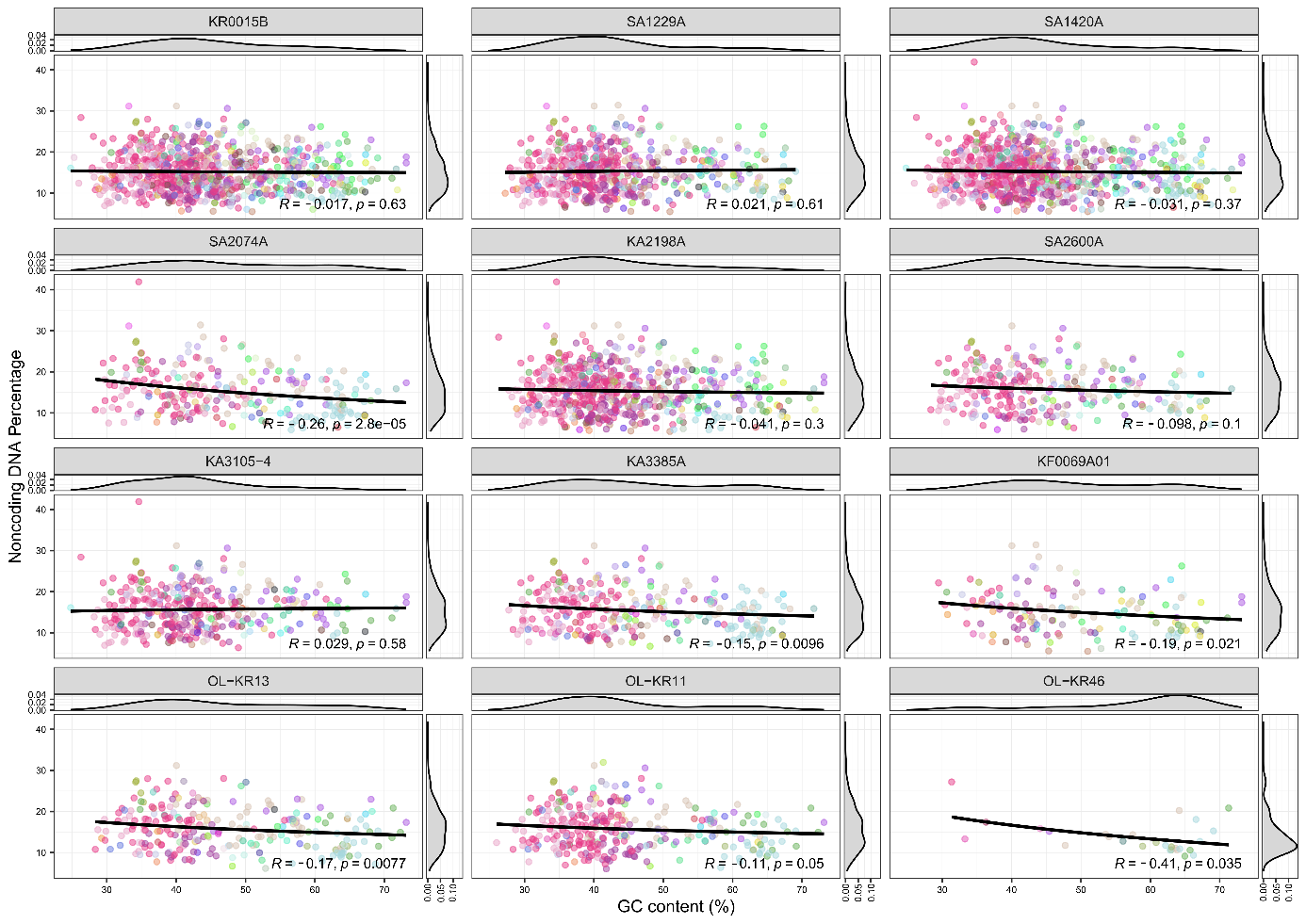
**

**Supplementary Figure S5:** Percentage of non-coding DNA in representative MAGs/SAGs in correlation with their GC content. Panels are ordered from left to right according to increasing sampling depth.

**
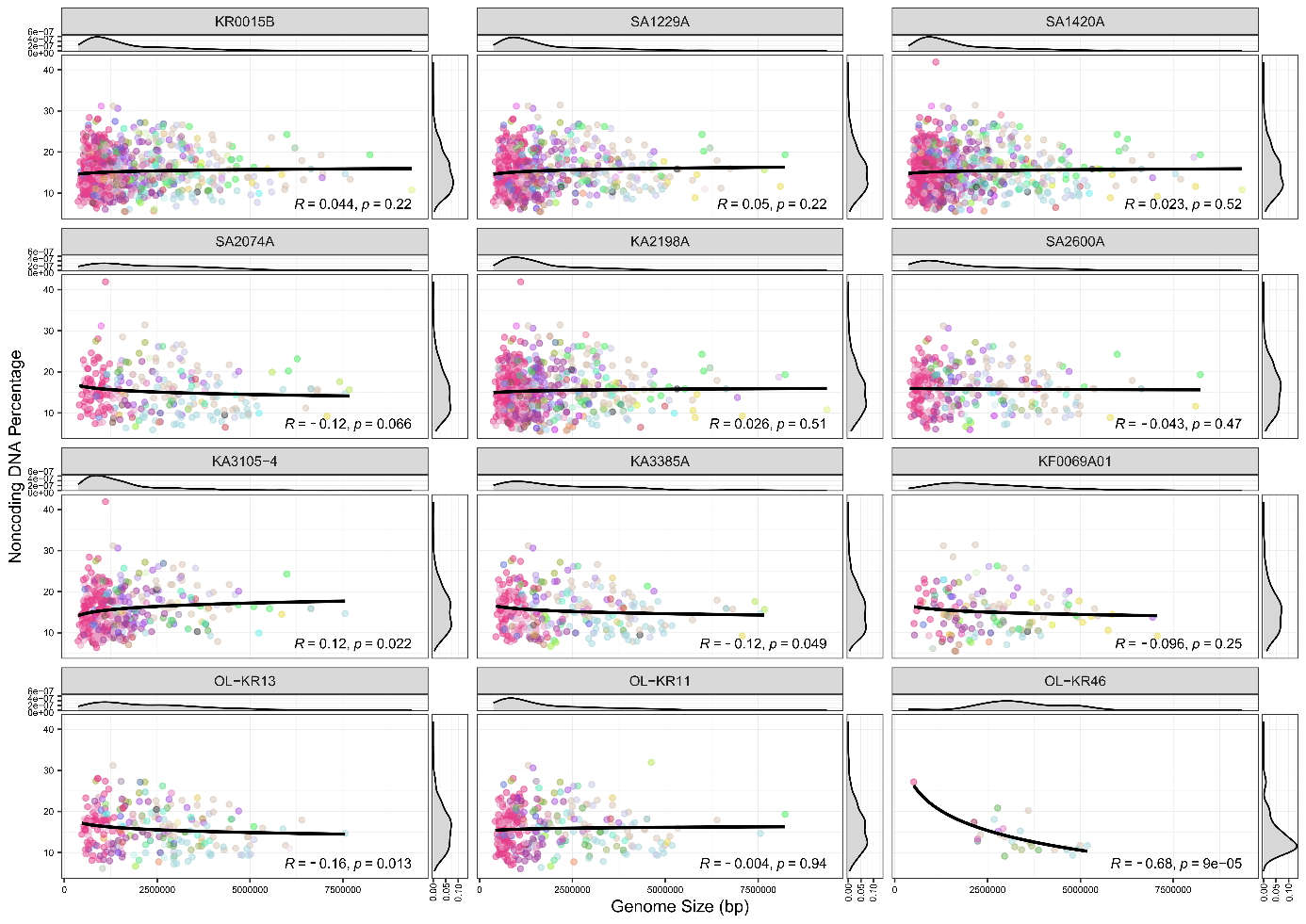
**

**Supplementary Figure S6:** Percentage of non-coding DNA in representative MAGs/SAGs in correlation with their genome size. Panels are ordered from left to right according to increasing sampling depth.

**
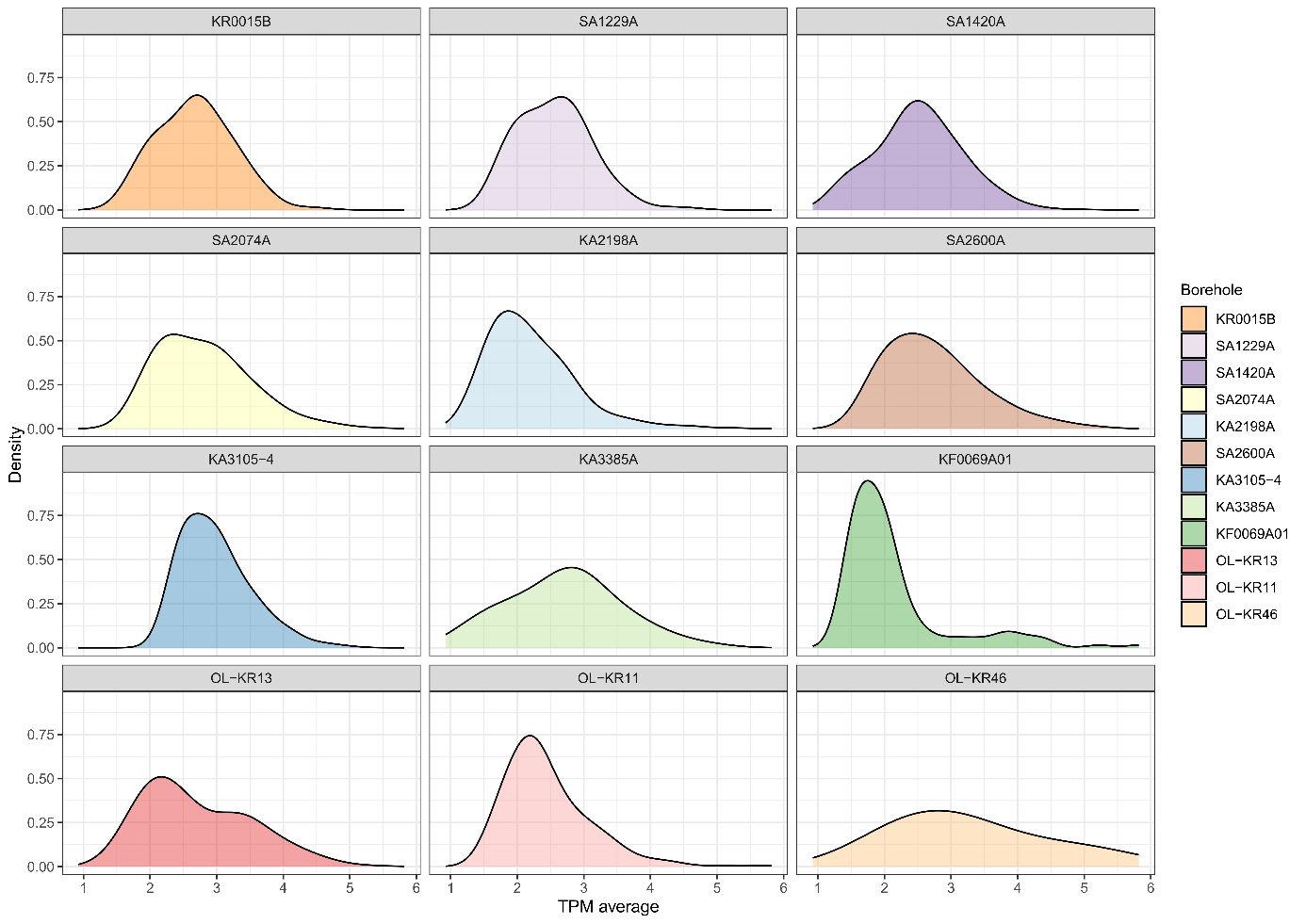
**

**Supplementary Figure S7:** Population size distribution of MAGs in different boreholes calculated as the average of the nonzero log_10_ TPM value of each representative MAG in all metagenomes sequenced for each borehole. Panels are ordered from left to right according to increasing sampling depth. Boreholes are color-coded consistently with Figure 1.

**
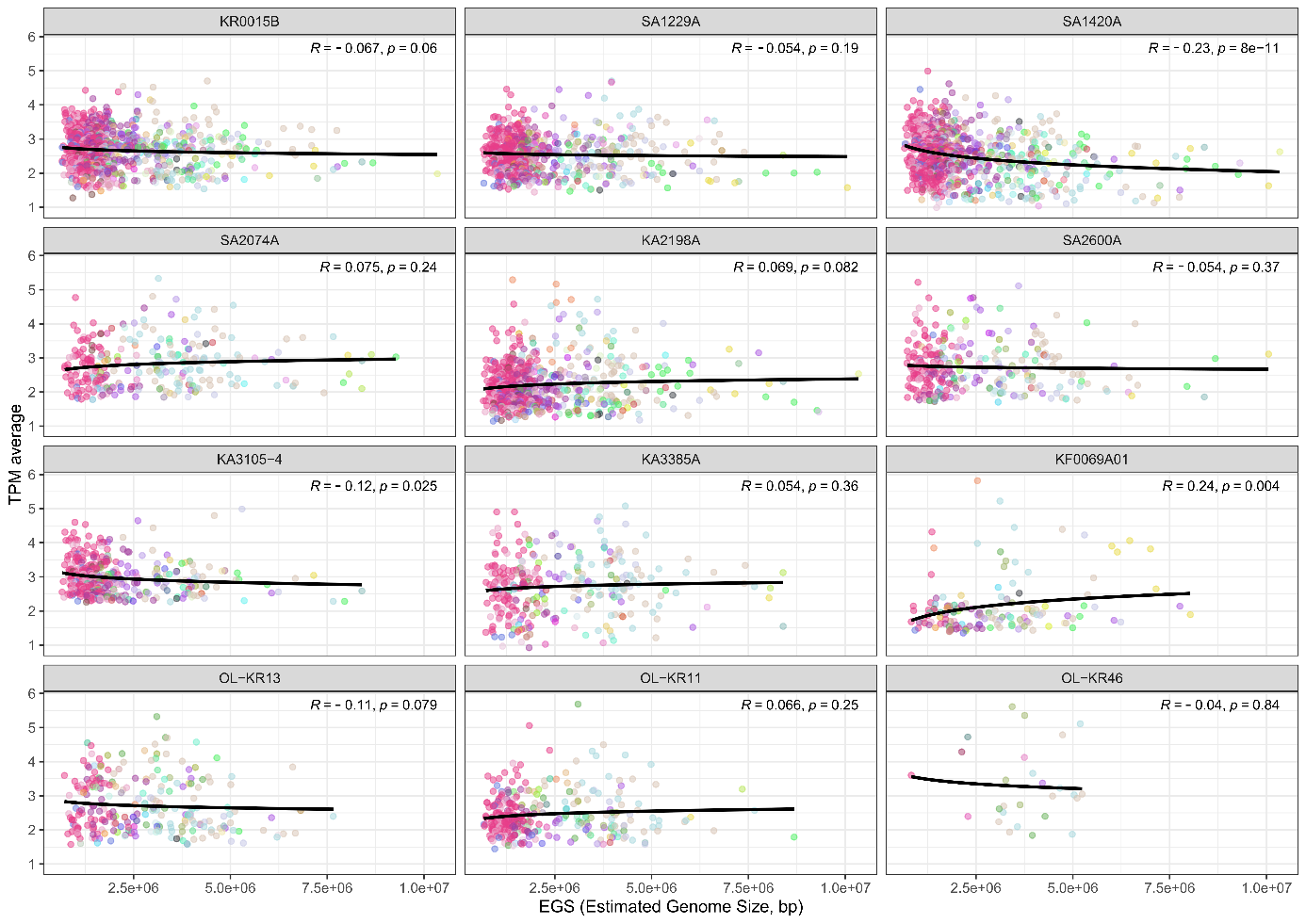
**

**Supplementary Figure S8:** Average abundance of representative MAGs/SAGs present in different boreholes across the range of estimated genome size (EGS). Panels are ordered from left to right according to increasing sampling depth.

**
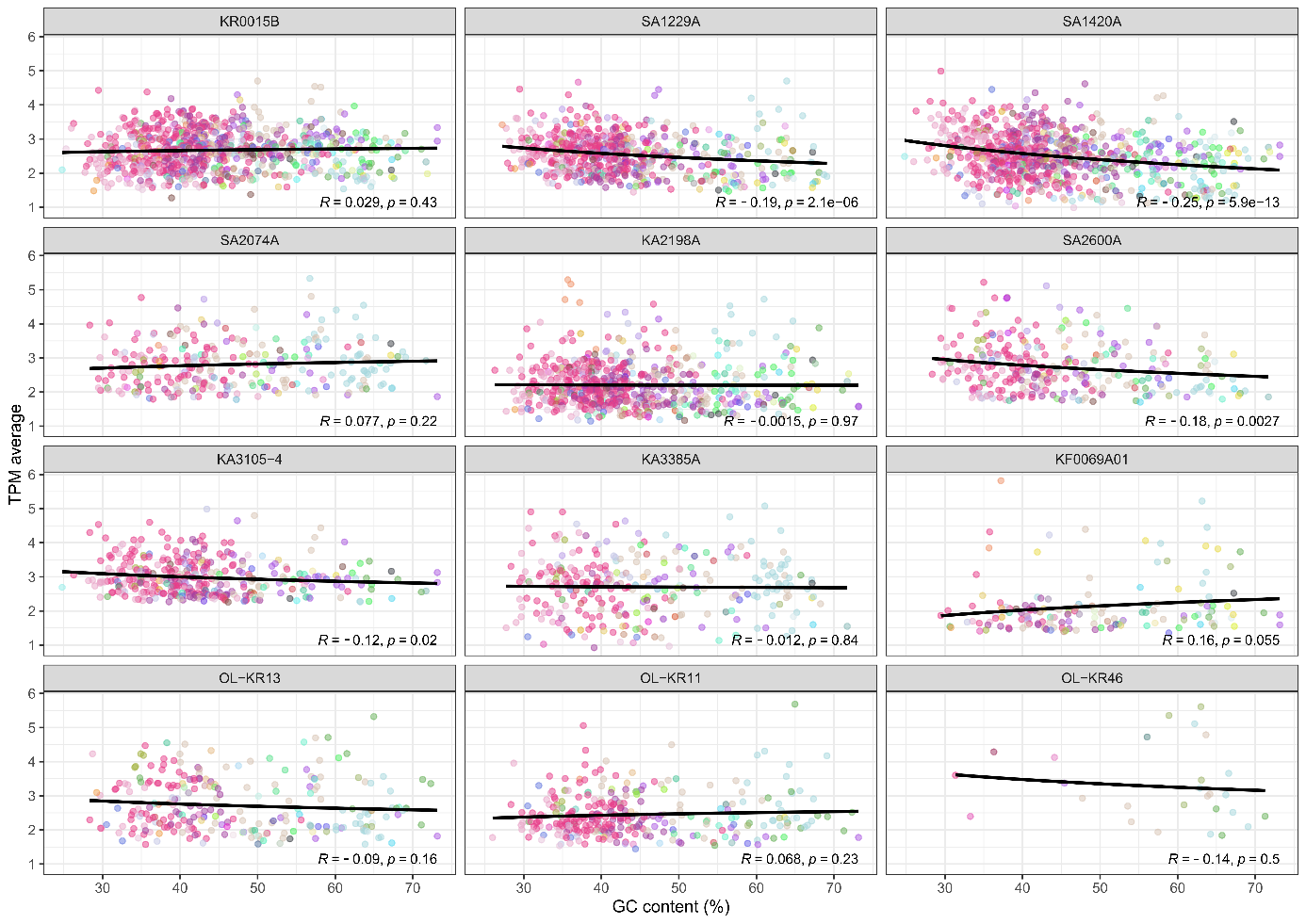
**

**Supplementary Figure S9:** Average abundance of representative MAGs/SAGs present in different boreholes across the range of GC content. Panels are ordered from left to right according to increasing sampling depth.

**
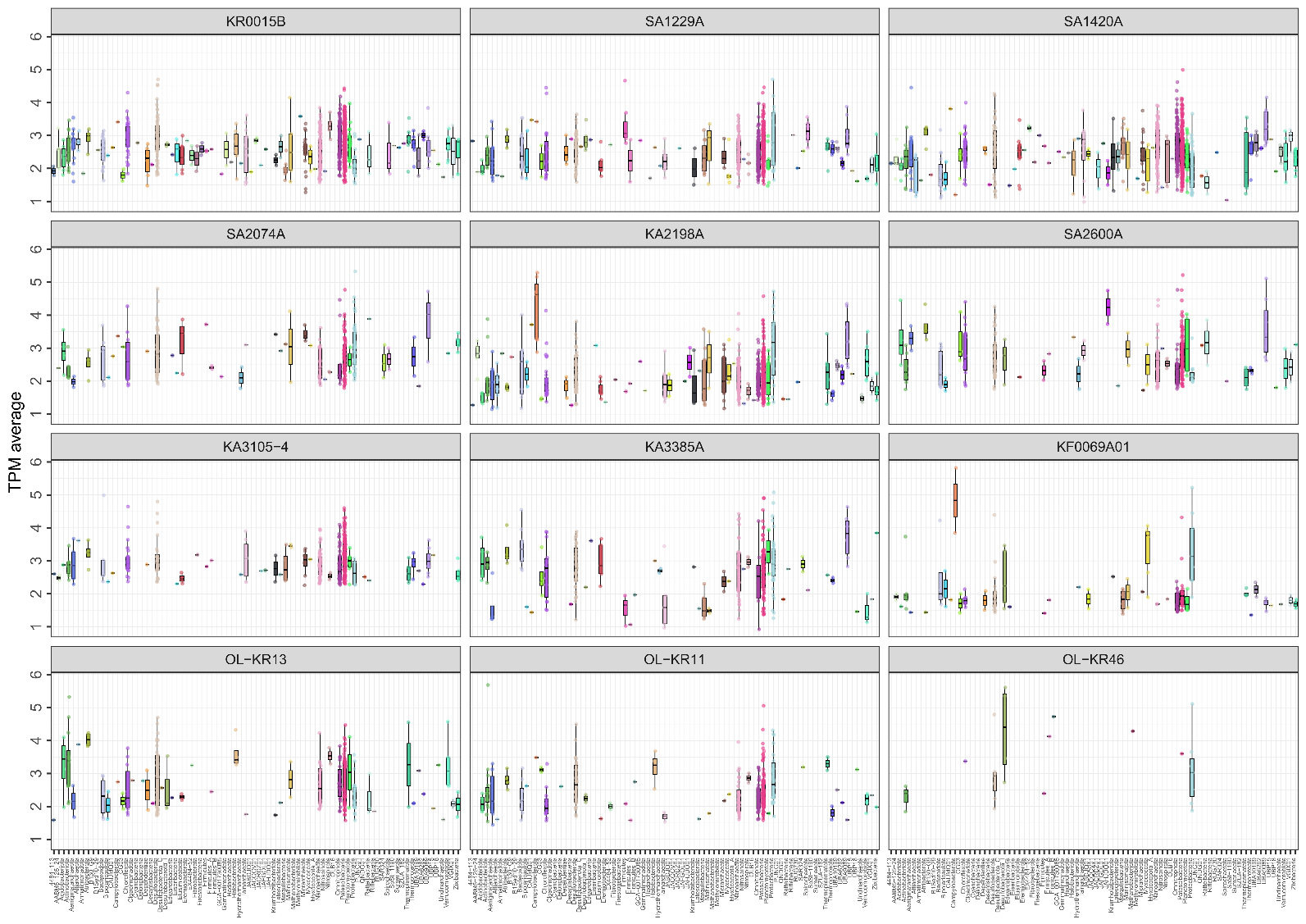
**

**Supplementary Figure S10:** Distribution of population size for different phyla present in the boreholes calculated as the average of the nonzero log_10_ TPM value of each representative MAG in all metagenomes sequenced for each borehole. Panels are ordered from left to right according to increasing sampling depth.


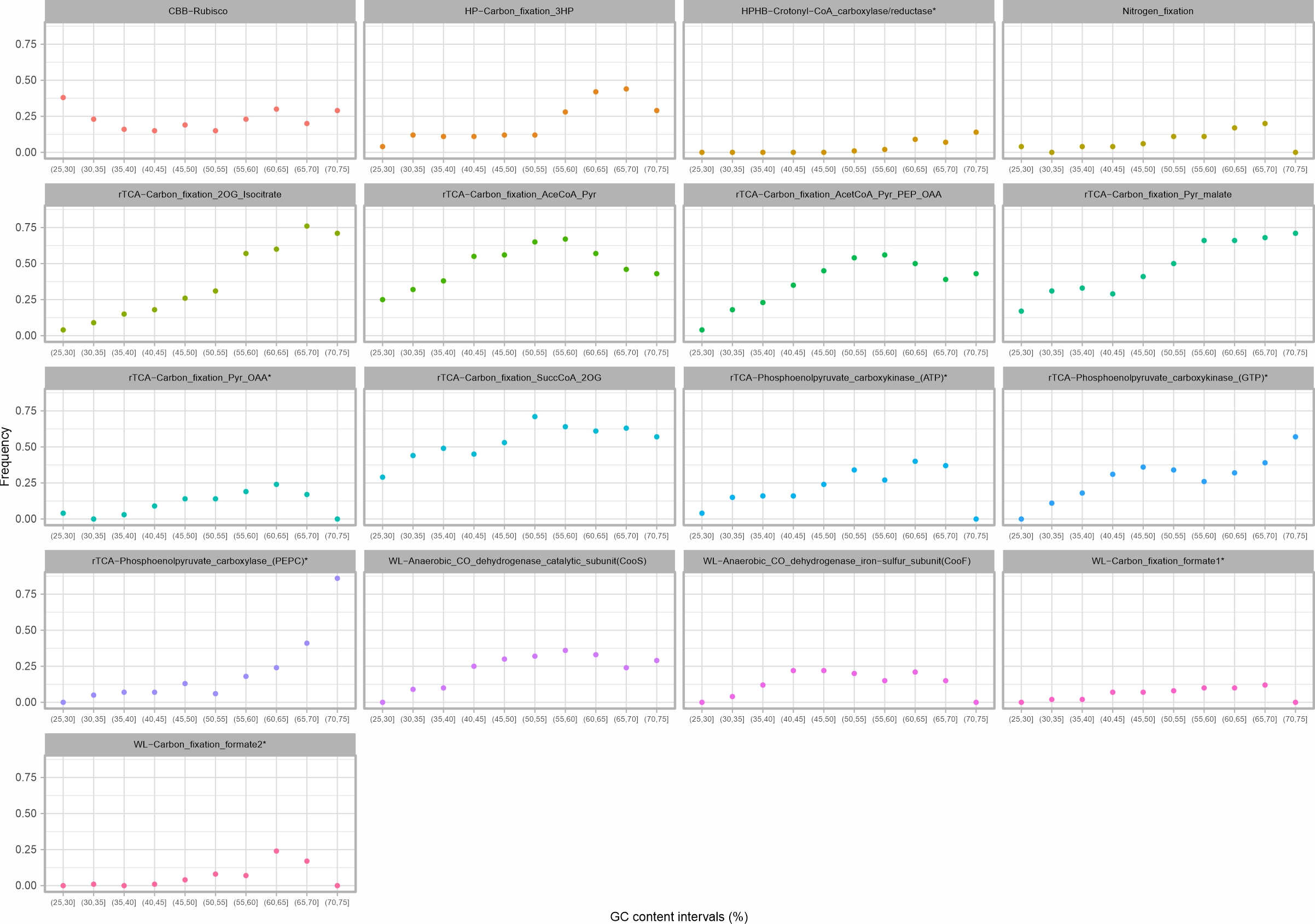


**Supplementary Figure S11:** Frequency of MAGs/SAGs containing genes for different carbon and nitrogen fixation pathways across the range of GC content. The number of MAGs/SAGs containing genes for each pathway was counted in intervals of 5% GC content and then normalized by the number of all MAGs/SAGs present in each interval.


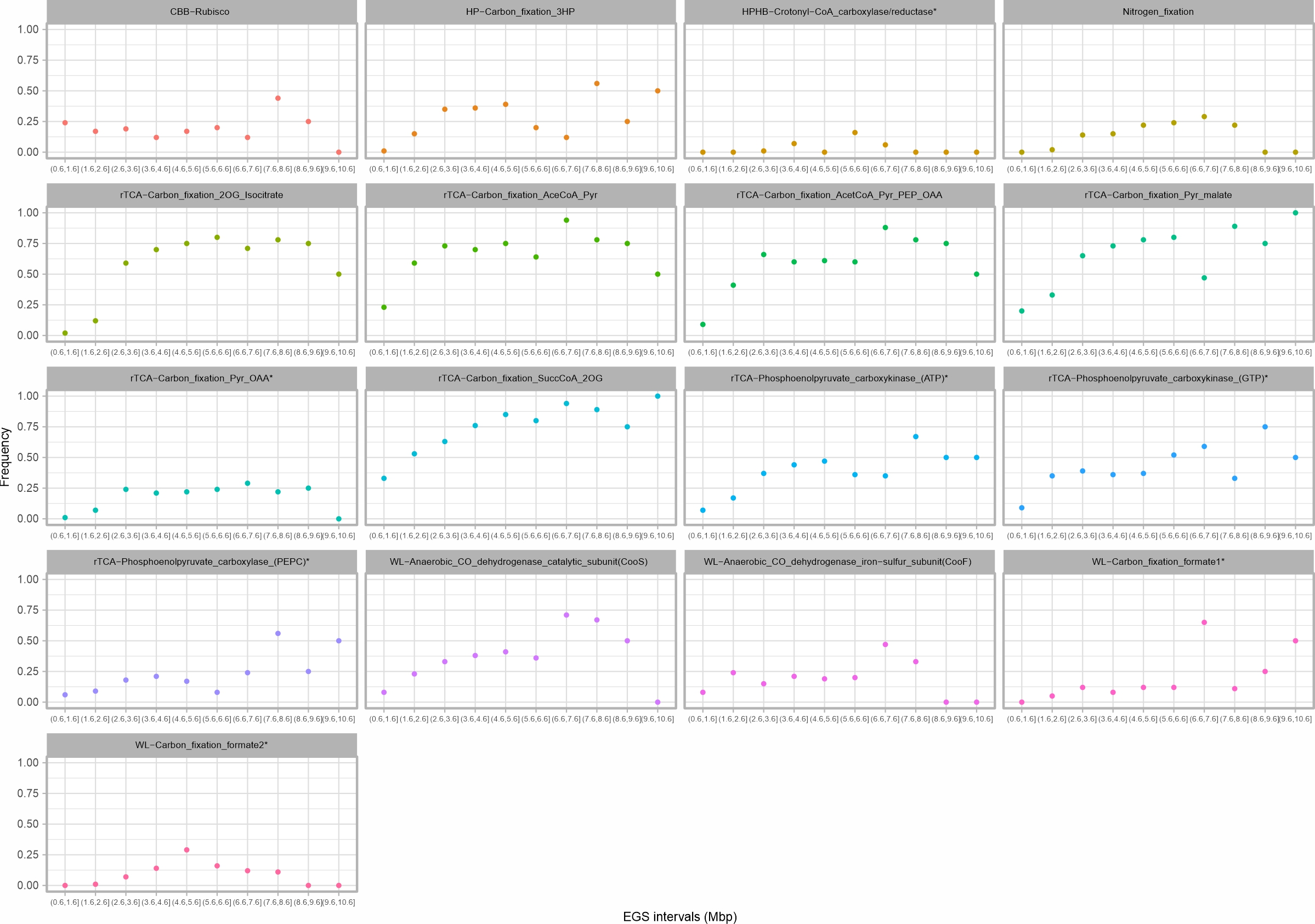


**Supplementary Figure S12:** Frequency of MAGs/SAGs containing genes for different carbon and nitrogen fixation pathways across the range of estimated genome size. The number of MAGs/SAGs containing genes for each pathway was counted in intervals of 1 Mbp genome size and normalized by the number of all MAGs/SAGs present in each interval.

**
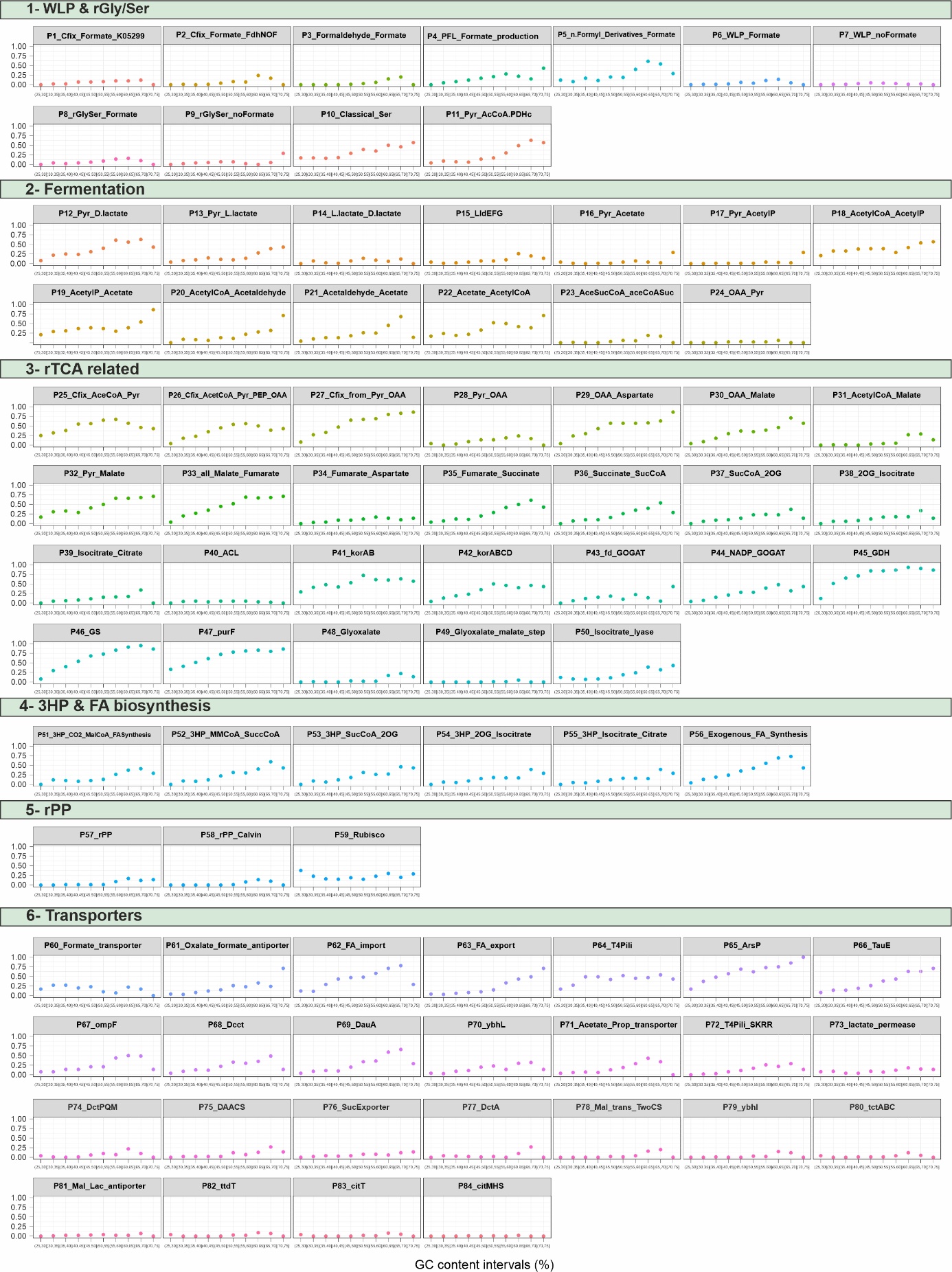
**

**Supplementary Figure S13:** Frequency of MAGs/SAGs containing genes for 58 modules in carbon fixation metabolism as well as genes encoding 25 transporters across the range of GC content. The number of MAGs/SAGs containing genes for each module was counted in intervals of 5% GC content and normalized by the number of all MAGs/SAGs present in each interval.


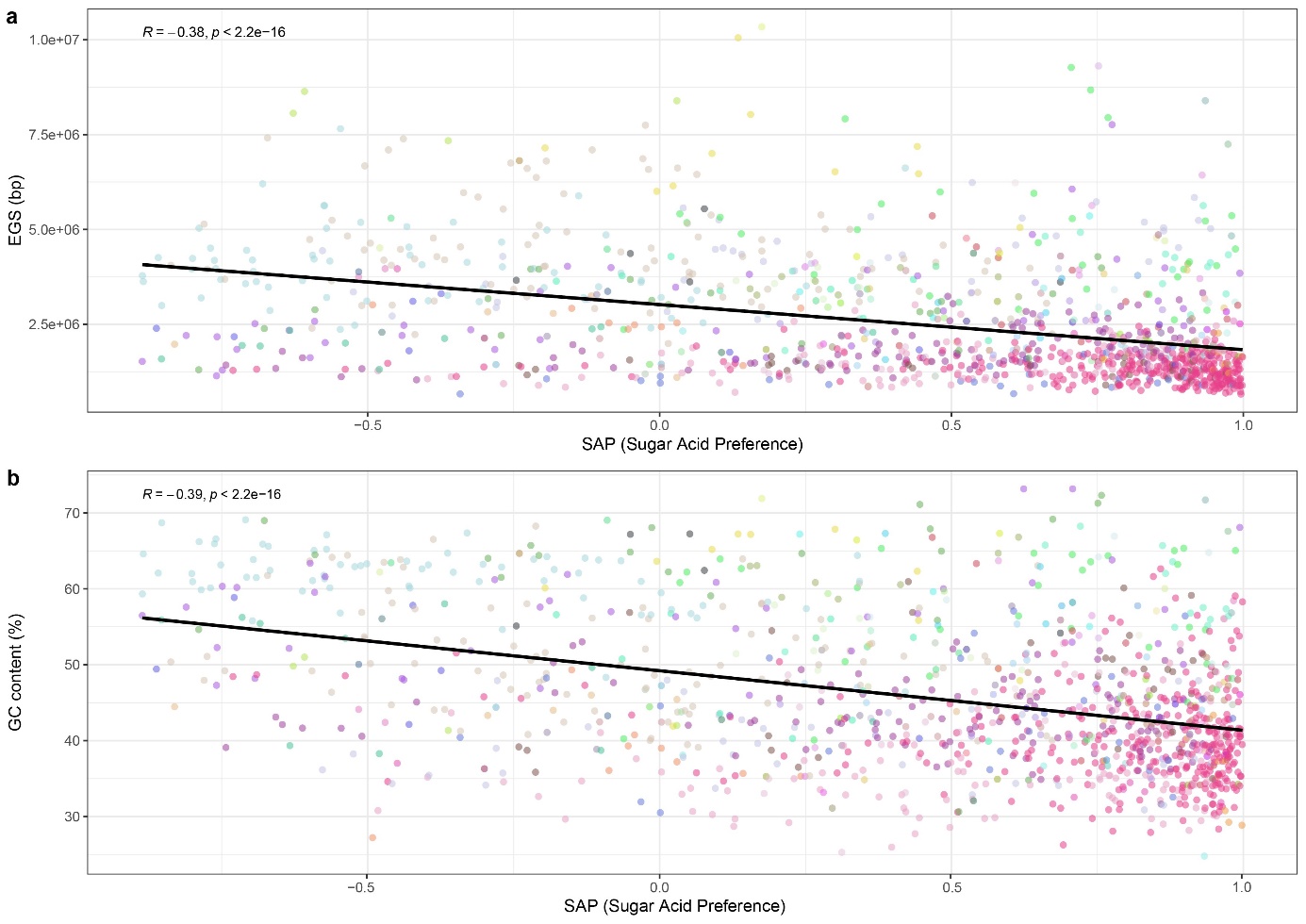


**Supplementary Figure S14:** Distribution of calculated SAP (sugar acid preference) index across the range of GC content (a) and estimated genome size (EGS) (b).


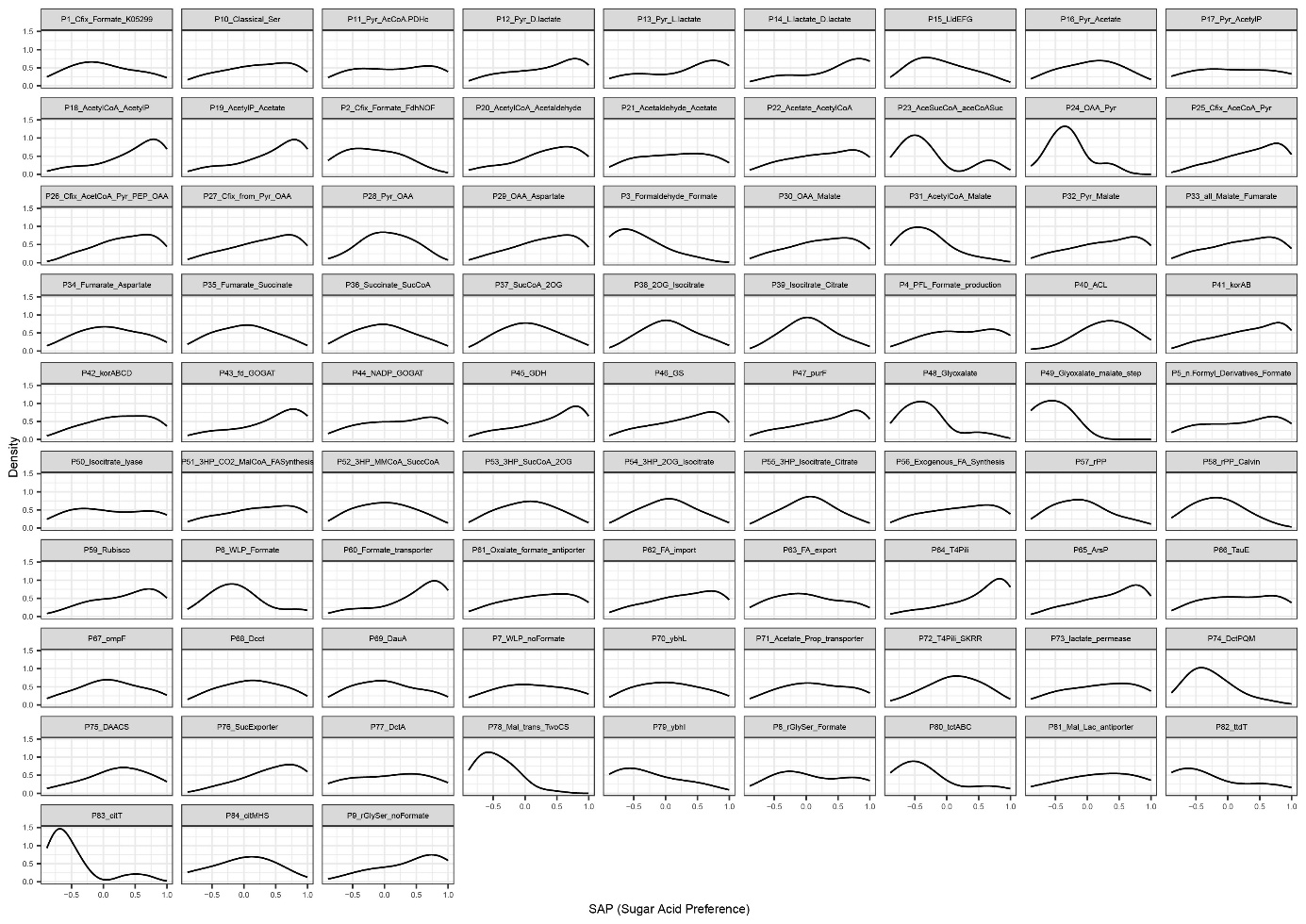


**Supplementary Figure S15:** Frequency of MAGs/SAGs encoding genes for 58 modules involved in carbon metabolism as well as genes encoding 25 transporters across the range of sugar acid preference (SAP) index (ranging from 1, indicating sugar specialists, to -1, indicating acid specialists).

**
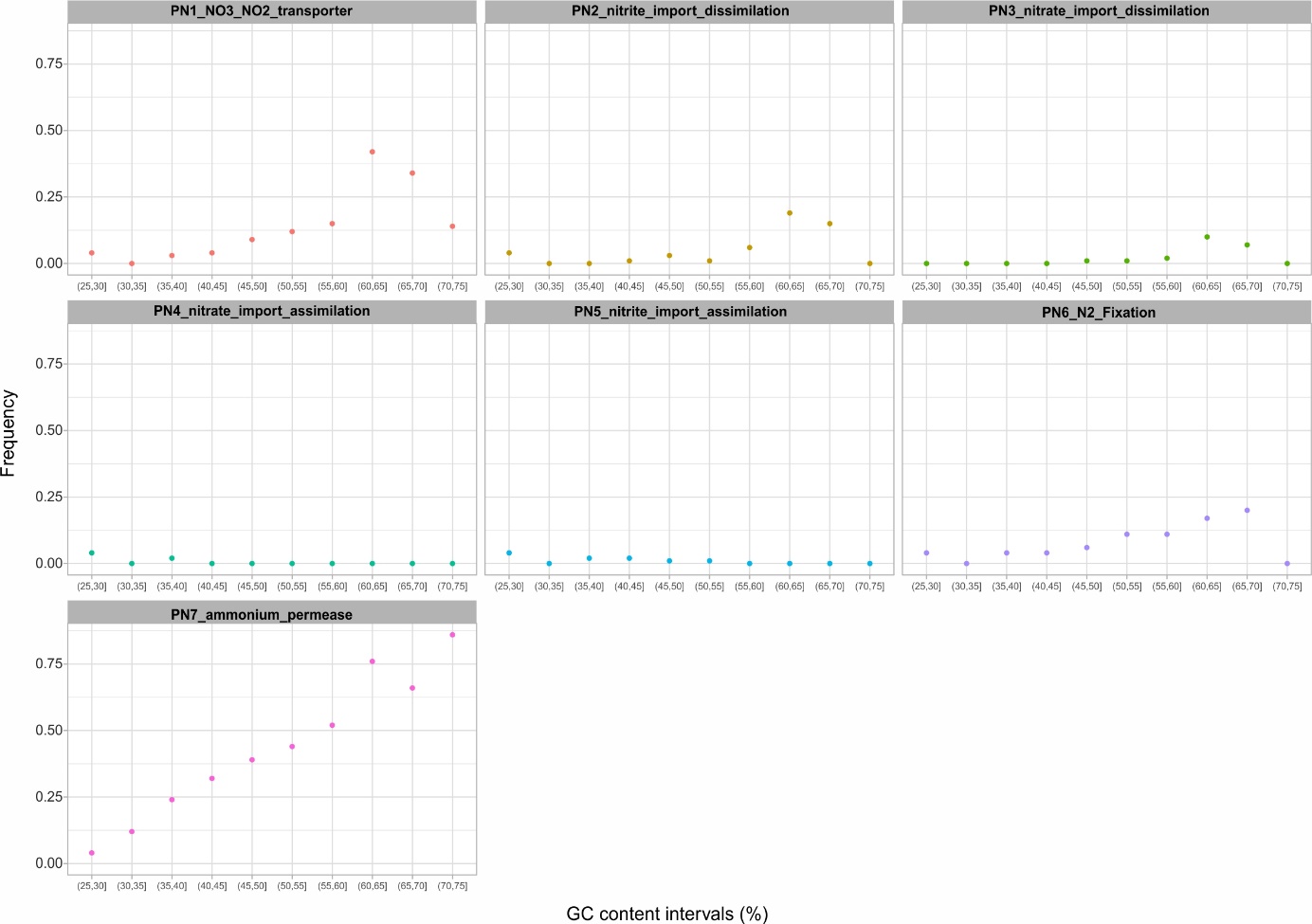
**

**Supplementary Figure S16:** Frequency of MAGs/SAGs containing genes for five modules in nitrogen metabolism as well as genes encoding two transporters across the range of GC content. The number of MAGs/SAGs containing genes for each module is counted in intervals of 5% GC content and normalized by the number of all MAGs/SAGs present in each interval.


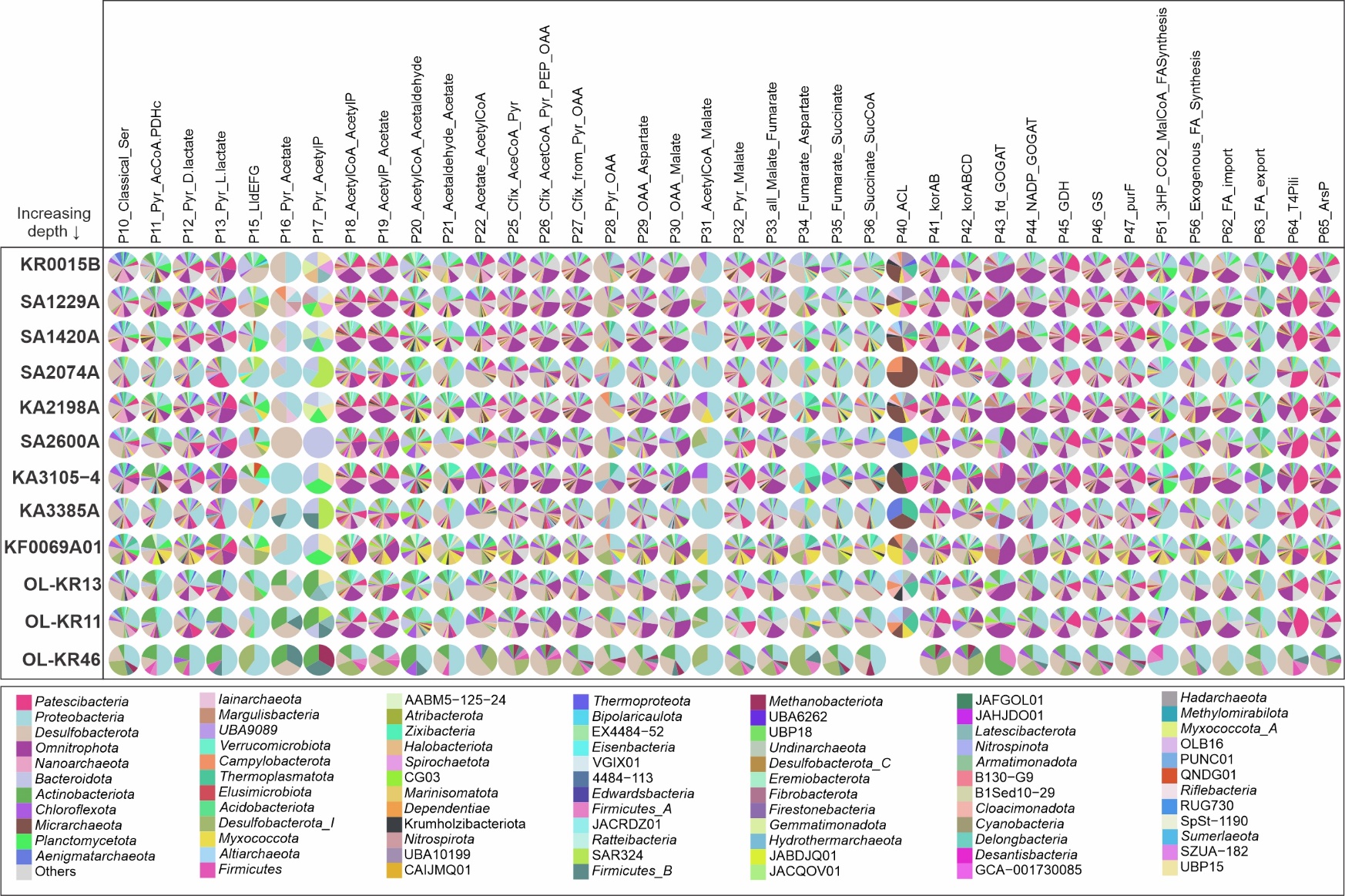


**Supplementary Figure S17:** Composition of microbes carrying genes for each core metabolic module in different boreholes. The community of microbes encoding each metabolic module in different boreholes is shown as a pie chart. The color legend at the bottom of the plot shows taxonomic affiliation of MAGs/SAGs encoding different modules at phylum level. Phyla with overall abundance lower than 1% in all boreholes were clumped together as “others” category.

**
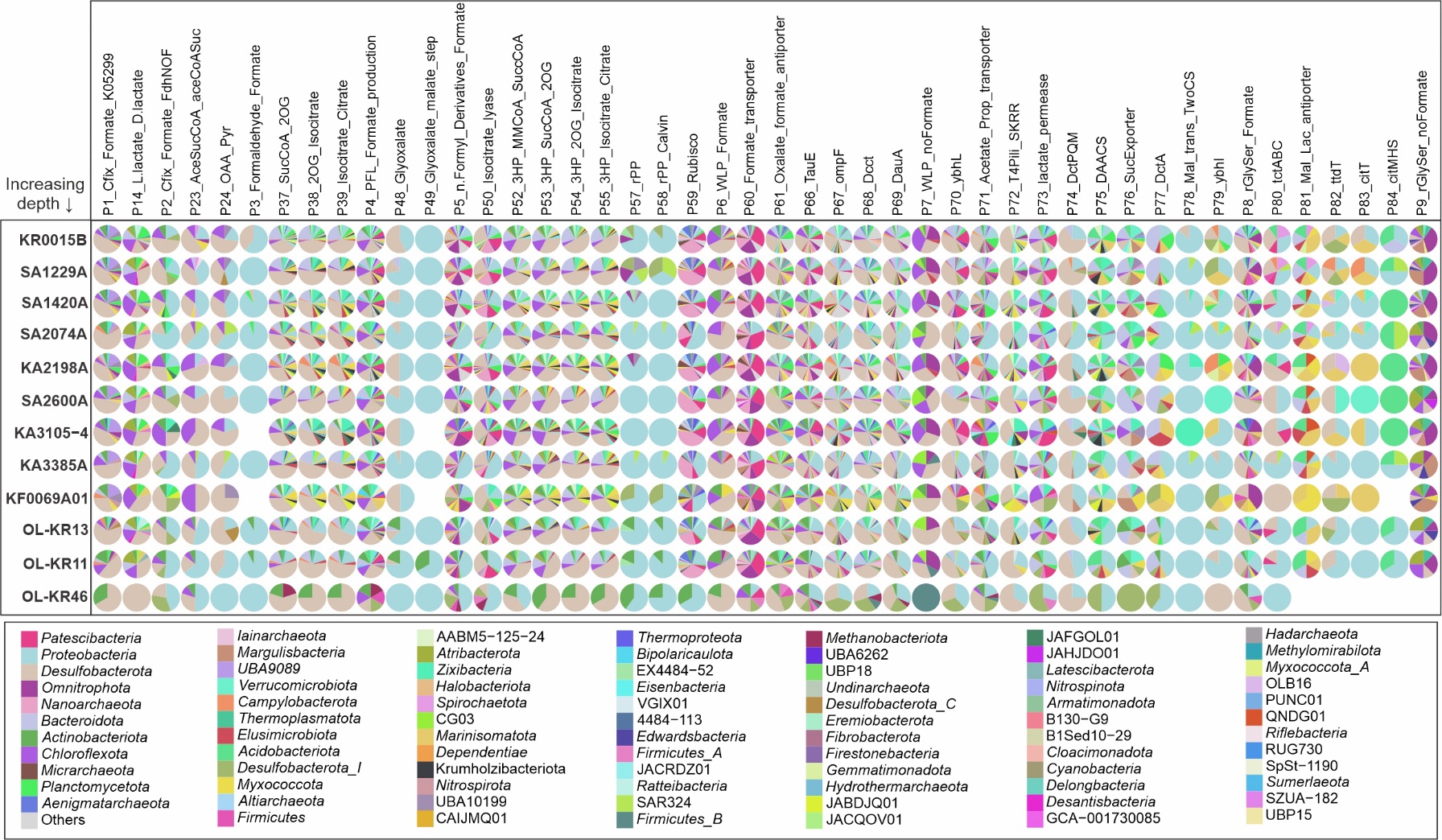
**

**Supplementary Figure S18:** Composition of microbes carrying genes for each metabolic module (other than those in the core) and transporters in different boreholes. The community of microbes encoding each metabolic module in different boreholes is shown as a pie chart. The color legend at the bottom of the plot shows the taxonomic affiliation of MAGs/SAGs encoding different modules at the phylum level. the number of MAGs/SAGs encoding phyla with overall abundance lower than 1% in all boreholes were clumped together as “others” category.


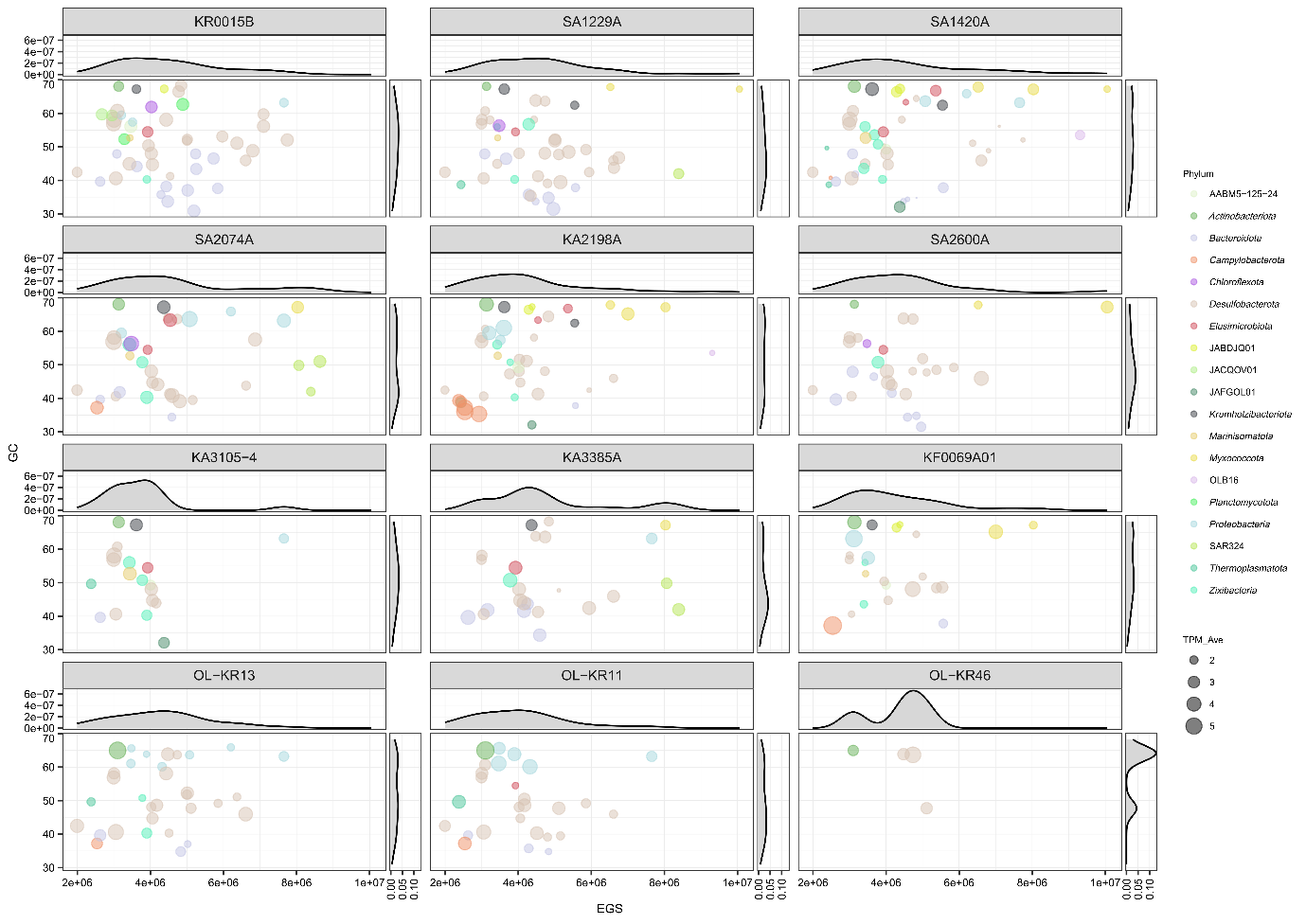


**Supplementary Figure S19:** Correlation between GC content and estimated genome size (EGS) of MAGs/SAGs encoding rTCA (encoding at least two of the three key genes) in different boreholes. The color of the circles represents different phyla, while their size indicates the average TPM (calculated as the average of the nonzero log_10_ TPM values of each rTCA-bearing MAG across all metagenomes sequenced for each borehole). Panels are ordered from left to right according to increasing sampling depth.
